# TRAP-seq Profiling and RNAi-Based Genetic Screens Identify Conserved Glial Genes Required for Adult Drosophila Behavior

**DOI:** 10.3389/fnmol.2016.00146

**Published:** 2016-12-22

**Authors:** Fanny S. Ng, Sukanya Sengupta, Yanmei Huang, Amy M. Yu, Samantha You, Mary A. Roberts, Lakshmanan K. Iyer, Yongjie Yang, F. Rob Jackson

**Affiliations:** Department of Neuroscience, Sackler Program in Biomedical Sciences, Tufts University School of MedicineBoston, MA, USA

**Keywords:** glia, translational profiling, Drosophila, behavior, circadian rhythm, locomotor activity

## Abstract

Although, glial cells have well characterized functions in the developing and mature brain, it is only in the past decade that roles for these cells in behavior and plasticity have been delineated. Glial astrocytes and glia-neuron signaling, for example, are now known to have important modulatory functions in sleep, circadian behavior, memory and plasticity. To better understand mechanisms of glia-neuron signaling in the context of behavior, we have conducted cell-specific, genome-wide expression profiling of adult Drosophila astrocyte-like brain cells and performed RNA interference (RNAi)-based genetic screens to identify glial factors that regulate behavior. Importantly, our studies demonstrate that adult fly astrocyte-like cells and mouse astrocytes have similar molecular signatures; in contrast, fly astrocytes and surface glia—different classes of glial cells—have distinct expression profiles. Glial-specific expression of 653 RNAi constructs targeting 318 genes identified multiple factors associated with altered locomotor activity, circadian rhythmicity and/or responses to mechanical stress (bang sensitivity). Of interest, 1 of the relevant genes encodes a vesicle recycling factor, 4 encode secreted proteins and 3 encode membrane transporters. These results strongly support the idea that glia-neuron communication is vital for adult behavior.

## Introduction

In mammals and insects, glial cells physiologically modulate neurons of the mature brain to regulate behavior and plasticity (Parpura and Zorec, 2010; Araque et al., 2014; Haydon and Nedergaard, 2015; Jackson et al., 2015; Zorec et al., 2015; Zwarts et al., 2015). Importantly, certain classes of adult Drosophila glial cells, including astrocytes, have morphological and molecular similarities to their mammalian counterparts (Awasaki et al., 2008; Doherty et al., 2009; Stork et al., 2012; Freeman, 2015; Omoto et al., 2015). In both Drosophila and mammals, individual astrocytes have processes that cover a relatively large territory, with the potential to regulate the activities of many different neuronal synapses. Other fly glial types are also components of so-called “tripartite” synapses that regulate neurotransmission (Danjo et al., 2011; Strauss et al., 2015). These features make Drosophila an attractive genetic model for the *in vivo* analysis of glia-neuron communication. Drosophila glia-neuron interactions are known to be important for development of the fly nervous system as well as normal and pathological neuronal degeneration that occurs in the adult brain (Doherty et al., 2009; Miller et al., 2012; Petersen et al., 2012; Hakim et al., 2014; Tasdemir-Yilmaz and Freeman, 2014), but only recently has it been documented that such interactions are important for adult behavior. Recent studies, for example, show that glia-neuron signaling in the Drosophila brain is an important component of circuit interactions that control neuronal excitability (Melom and Littleton, 2013; Rusan et al., 2014), circadian behavior (Ng et al., 2011; Jackson et al., 2015), sleep (Seugnet et al., 2011; Chen et al., 2015), olfaction (Liu et al., 2014), vision (Borycz et al., 2012; Rahman et al., 2012; Xu et al., 2015) and memory formation (Yamazaki et al., 2014; Matsuno et al., 2015). Given these findings, it is of interest to define glial factors, including secreted proteins, which mediate communication with neurons.

Peptides and proteins are secreted from astrocyte dense core vesicles (Verkhratsky et al., 2016) and regulate the differentiation and maintenance of synapses (Christopherson et al., 2005; Allen et al., 2012; Clarke and Barres, 2013; Singh et al., 2016). Mammalian astrocytic factors such as Thrombospondin, Glypicans, SPARC, and Hevin, for example, are known to regulate synaptogenesis (Christopherson et al., 2005; Kucukdereli et al., 2011; Allen et al., 2012), and it was recently shown, for example, that Hevin is secreted from thalamocortical astrocytes to promote assembly of glutamatergic synapses by bridging neurexin-neuroligin interactions (Singh et al., 2016). Less well understood are the functions of secreted glial proteins in the regulation of behavioral state in mammals and other models, although much is known about small molecules (gliotransmitters) that regulate adult behavior and plasticity (Zorec et al., 2016).

In the present report, we describe experiments employing Translating Ribosome Affinity Purification (TRAP) procedures, which we adapted to the Drosophila model (Huang et al., 2013), to define the genome-wide expression profile of adult fly astrocytes. With that dataset as a starting point, we have performed RNA interference (RNAi)-based genetic screens to identify proteins participating in glia-neuron signaling that is relevant for behavioral regulation. These studies have identified multiple secreted factors as well as glial transporters with roles in the regulation of neuronal excitability, locomotor activity and circadian behavior.

## Materials and methods

### Affinity purification of translating RNA from Drosophila astrocytes

We utilized methods identical to our published studies (Huang et al., 2013, 2014, 2015) to isolate ribosomes and translating RNAs from adult fly astrocytes; the protocols are outlined in detail in a recent review (Jackson et al., 2015). The current study utilized a Gal4 driver (*alrm-Gal4*) that expresses in fly astrocytes but not in CNS neurons or other glial cell types (Doherty et al., 2009) to drive expression of a characterized *UAS-EGFP-L10a* transgene (Huang et al., 2013; encoding an EGFP-tagged L10a ribosomal subunit). To be certain EGFP-L10a was not expressed in non-CNS neurons (e.g., photoreceptors of the head), *alrm-Gal4*>*UAS-EGFP-L10a* flies also carried one copy of *elav-Gal80* (resulting in pan-neuronal inhibition of Gal4 expression). Standard crosses were performed to generate progeny carrying one copy each of the three transgenes. These flies were entrained to a light-dark cycle consisting of 12 h of light and 12 h of dark (LD 12:12) for at least 4 days and then flash frozen at Zeitgeber Time 1 (ZT1) of the diurnal cycle. Head tissues were collected using standard sieving procedures.

For TRAP analysis, approximately 200 heads were homogenized and the tissue lysate cleared and processed as described in Huang et al. (2013). About 1/10 of the cleared lysate was retained for extraction of head total RNA. The remaining lysate was processed according to our published methods. Importantly, a high affinity anti-GFP antibody was employed for TRAP (HtzGFP-19C8; Sloan Kettering Antibody & Bioresource Core Facility). Typically, about 30 ng of RNA was recovered by TRAP from 200 heads. RNA fractions from multiple TRAP experiments were pooled to obtain two independent samples of about 200 ng each. Similarly, two independent total RNA samples of ~200 ng each were prepared. The TRAP and total RNA samples were then used to generate RNA-seq libraries using the Illumina TruSeq RNA kit (v2). RNAs were poly-A selected, fragmented and reverse transcribed into double-stranded cDNAs using random primers. The double-stranded cDNAs were then ligated with Illumina adaptors and PCR amplified. The resulting libraries were purified and subjected to RNA-seq analysis.

### RNA-seq analysis of fly TRAP libraries

Drosophila RNA-seq libraries were sequenced using an Illumina HiSeq 2000 instrument of the Tufts University Core Facility (TUCF). Single-end, 100-base sequence reads were obtained and their quality analyzed using the quality control metrics provided by the FastQC pipeline (http://www.bioinformatics.babraham.ac.uk/projects/fastqc/). We obtained, on average, about 15 million high-quality reads for all samples after removing low-quality reads. Fastq files The STAR (Dobin et al., 2013) and the HTSeq (Anders et al., 2014) algorithms were employed to map and identify genes expressed in astrocytes (shown in Table 1). Directionality of transcription was not considered in the analysis due to the unstranded nature of the RNA-seq library preparation. Later studies used Tophat2 (v 2.0.8) and Bowtie2 (v 2.1.0) (Trapnell et al., 2009; Langmead and Salzberg, 2012) to map reads; using these algorithms, an average of 84% of the high-quality reads could be mapped to the Drosophila 5.22 reference genome. The Cuffdiff2 algorithm (Trapnell et al., 2013) was used to identify significant changes in mRNA expression between the total and astrocyte-enriched samples. Samples were analyzed to identify genes for which the average expression (FPKM value) was greater in TRAP-derived vs. total RNA. Using a *q*-value of <0.05, 1236 genes were considered to have astrocyte enriched expression.

**Table 1:** **RNAi transgenes with effects on activity level**.

**Strain^δ^**	**Gene^α^**	**n**	**Activity ± SEM^β^**	**Human Homolog**
*repo-Gal4>UAS-30731*	*Treh*^γ^	31	10.7 ± 0.7^**^	*Treh*
*UAS-30731*		30	31.0±1.2	
*repo-Gal4*		32	25.8±1.0	
*repo-Gal4>UAS-30730*^λ^	*Treh*	10	4.0 ± 0.7^***^	
*UAS-30730*		16	27.0±1.7	
*repo-Gal4*		12	16.9±1.2	
*repoGal4>UAS-31203*	*X16*^γ^	14	37.5 ± 2.8^***^	*Srsf7*
*UAS-31203*		16	26.1±1.9	
*repo-Gal4*		12	16.5±1.2	
*repo-Gal4 >UAS-100721*	*Tsp*^γ^	16	18.6 ± 2.0^***^	*Tsp*
*UAS-100721*		16	27.5±1.6	
*repo-Gal4*		12	32.1±2.8	
*repo-Gal4>UAS-7535*	*Tsp*	16	19.1 ± 1.1^**^	
*UAS-7535*		11	29.8±2.8	
*repo-Gal4*		12	32.1±2.8	
*repo-Gal4>UAS-36876*	*4EHP*^γ^	28	18.5 ± 1.2^**^	*EIF4E2*
*UAS-36876*		47	29.6±1.4	
*repo-Gal4*		29	26.8±1.6	
*repo-Gal4>UAS-43990*	*4EHP*	30	11.8 ± 0.9^**^	
*UAS-43990*		27	23.0±1.6	
*repo-Gal4*		29	26.8±1.6	
*repo-Gal4>UAS-51149*	*Spase25*^γ^	16	19.4 ± 0.7^**^	*Spcs2*
*UAS-51149*		16	34.9±4.5	
*repo-Gal4*		16	32.09±3.0	
*repo-Gal4>UAS-13538*	*CG1537*	20	15.4 ± 3.2^**^	–
*UAS-13538*		16	30.9±3.2	
*repo-Gal4*		32	29.1±1.4	
*repo-Gal4>UAS-102531*	*CG1537*	13	13.4 ± 1.0^**^	
*UAS-102531*		15	25.1±1.1	
*repo-Gal4*		16	29.9±1.4	
*repo-Gal4>UAS-48550*	*CG1552*^ϕ^	16	16.4 ± 0.9^*^	–
*UAS-48550*		10	26.6±2.6	
*repo-Gal4*		16	23.4±1.8	
*repo-Gal4>UAS-1552R-1*	*CG1552*	16	17.7 ± 1.3^*^	
*UAS-1552R-1*		16	23.7±2.7	
*repo-Gal4*		12	32.1±2.8	
*repoGal4>UAS-43017*	*CG14141*^ϕ^^,^ ^π^	41	50.9 ± 3.0^**^	–
*UAS-43017*		49	24.8±1.0	
*repo-G4*		38	19.8±1.0	

α *All genes show astrocyte enriched expression except for 3 genes: Spase25, which is expressed in TRAP samples at a level higher than that observed in the total lysate; Tsp which has approximately the same expression level in TRAP and total lysate samples; and 4EHP which is expressed at low levels (~100–250 reads) in both TRAP and total lysate samples*.

β *Average activity/30 min in LD*.

δ *All RNAi-expressing strains exhibited reduced or increased activity in both LD and DD*.

ϕ *Nervous system-specific expression*.

γ *Mouse ortholog has astrocyte expression*.

λ *Data from LD days 1–3*.

π *Only 1 RNAi line exists for this gene*.

* *p ≤ 0.05*;

** *p ≤ 0.01*;

*** *p ≤ 0.001. Bold values indicate experimental genotypes that differ significantly from controls*.

### Gene ontology (GO) analyses

GO analyses were performed using the Database for Annotation, Visualization, and Integrated Discovery (DAVID, version 6.8) annotation tools (https://david.ncifcrf.gov/) to determine overrepresented categories of GO biological processes (BPs). We used an equivalent number of fly and mouse genes (1236) for the comparative GO analyses; the mouse gene list represented 1236 genes with highest FPKM score from the average of two different astrocyte RNA-seq datasets (Zhang et al., 2014). Gene IDs were derived using FlyBase and the Mouse Genome Informatics Database (MGI) to convert fly and mouse gene symbols, respectively, to gene numbers (FBgns). According to DAVID, biological processes (BPs) were associated with 932 of the 1236 fly astrocyte-enriched genes and 1252 mouse genes from the Zhang et al. study (2014). We assume the total mouse list is larger than 1236, because gene symbols often correspond to multiple IDs. Significance (*p*-values) was calculated using Fisher's exact test and then corrected by two multiple-testing correction methods: Bonferroni (more stringent) and Benjamini (less stringent). The false discovery rate (FDR) was calculated using the Benjamini method. Only terms with *p* < 0.05 by the Benjamini method are included in Table S4.

### qPCR verification of RNA-seq results

To verify enrichment of mRNAs, ribosome-bound or total RNA was converted to cDNA using SuperScript II reverse transcriptase (Invitrogen) with random hexamers. The primers shown in Table S3 were employed for quantitative PCR (qPCR) using a Stratagene real-time cycler and SYBR green as a reporter for amplification. RNA abundance was analyzed using *rp49* as an internal control, and enrichment was expressed as a ratio of the TRAP to total RNA signal (Figure S1).

### Immunohistochemistry

Primary antibodies specific for Repo (mouse anti-repo 8D12; U. Iowa Hybridoma Center), GFP (rabbit HtzGFP-19C8, Sloan Kettering), PER and PDF were employed at dilutions of 1/500, 1/1000, 1/15,000, and 1/100, respectively, to detect the proteins in hand-dissected brain whole mounts. Cy3-labeled anti-mouse, Alexa647 anti-mouse, Alexa647 anti-rabbit, Alexa488 anti-mouse, and Alexa488 anti-rabbit secondary antibodies were employed at dilutions of 1/500 (Cy3 anti-mouse from Jackson ImmunoResearch; Alexa antibodies from Life Technologies). Antibody staining was performed as previously described (Ng et al., 2011). Confocal imaging was performed using Leica TCS SP2 AOBS, Leica SP8, or Nikon A1R microscopes. PDF staining in CG9657 RNAi and control flies was scored blindly by two independent observers who assessed cell bodies, the dorsal projections of the small ventral lateral clock neurons (s-LNvs), the contralateral projections of the large ventral lateral neurons (l-LNvs) within the posterior optic tract (POT) and the dendritic (optic lobe) projections of the l-LNv neurons. Cell and projections morphologies were scored using the following numerical system: 2, normal morphology; 1, obvious differences in morphology and/or PDF intensity; and 0, absence of visible PDF staining in cells or projections. Average scores are presented in Figure S3.

### Circadian behavioral analysis

Circadian locomotor activity was monitored using the Trikinetics Drosophila Activity Monitor (DAM) system, as previously described (Ng et al., 2011). Flies were briefly anesthetized with CO_2_, loaded in pyrex glass tubes, and placed in infrared monitors of the DAM system. They were entrained to LD 12:12 for 3–4 days prior to transfer to constant dark (DD) conditions. Activity data was collected in 30-min bins and analyzed using a MatLab-based circadian software package (Levine et al., 2002). This package uses correlogram analysis to compute circadian period and the robustness of rhythmicity. Average Rhythmicity Indices (RIs) were calculated from the third peak of the correlogram using values from the entire control or experimental populations. Statistical analysis of results was performed by initially assessing normality using the D'Agostino & Pearson omnibus normality test. If the data did not pass a normality test, we used the Kruskal-Wallis test (non-parametric ANOVA) with Dunn's Multiple Comparison test. One-way ANOVA with Tukey-Kramer Multiple Comparisons test was used if the data passed a normality test (GraphPad).

### Assaying bang sensitivity

To determine whether flies were sensitive to vibration (bang sensitive), tests were performed between ZT1 and ZT6. For all experiments, flies were transferred into empty vials, allowed to rest for 15–20 min, and then subjected to high speed vortexing for 10 s using a bench-top vortexer. A video camera was employed to capture the behavior of flies during vortexing and for 45 s afterwards. For the experiments with *nrv2*, the maximum number of flies that became paralyzed (dorsal side down or completely immobilized) was recorded at 10, 20, 30 and 45 s after vortexing (using the time slider function of VCL video software). For experiments with *Oat*, which were obviously debilitated prior to the test, flies were gently banged in vials containing medium.

## Results

### TRAP profiling fly astrocytes

**Figure 1:**
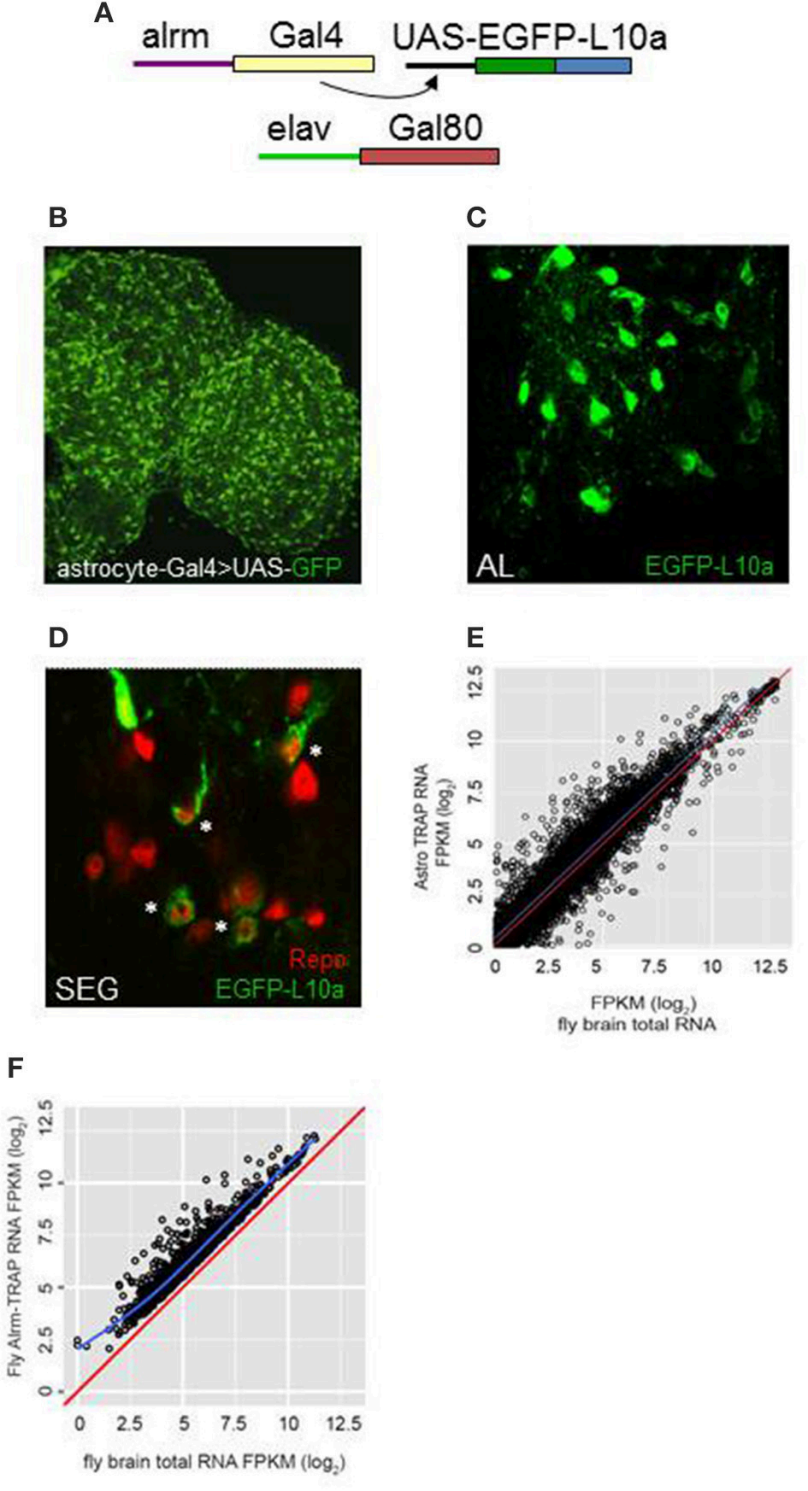
**TRAP analysis identified genes with enriched expression in astrocytes. (A)** The Gal4/UAS system was employed for astrocyte-specific expression. Alrm-Gal4 is expressed only in astrocytes of the central brain (Doherty et al., 2009). Flies also carried elav-Gal80 to inhibit Gal4 activity in neurons of the brain and ensure glia-specific expression. **(B)** Astrocytes of the adult brain, highlighted by expression of UAS-GFP, using the NP3233 Gal4 astrocyte driver. **(C,D)** Expression of UAS-EGFP-L10a in astrocytes of the antennal lobe (AL) or subesophageal ganglion (SEG). Note the cytoplasmic localization of signal in D in a subset of Repo-positive cells (^*^). **(E)** Comparison of FPKM values for all detected genes for TRAP-seq vs. total RNA-seq, illustrating enriched expression for many genes (those above the blue line). The red line in this and next panel indicates a hypothetical correlation of 1 for TRAP vs. total RNA samples. **(F)** Comparison of FPKM values for astrocyte-enriched genes in TRAP-seq vs. total RNA-seq samples, illustrating enrichment for more than 1200 genes. The blue line indicates the average correlation for all genes.

We employed TRAP techniques (Heiman et al., 2008; Huang et al., 2013) and flies expressing an EGFP-tagged L10a ribosomal subunit to carry out expression profiling of adult fly CNS astrocytes (Figure 1A; see Section Materials and Methods). The *alrm-Gal4* driver used in these experiments is expressed in astrocyte-like glia of the fly nervous system (Figure 1B; Doherty et al., 2009). As shown in Figure 1, the tagged L10a subunit was detected in the cytoplasm of glial cells in *alrm-Gal4*>*EGFP-L10a; elav-Gal80* flies (Figure 1C) and importantly in a subset of Repo-positive glial cells (Figure 1D), consistent with astrocyte expression. The *elav-Gal80* transgene was included in the genetic background to ensure that EGFP-L10a was not expressed in neurons. Tagged ribosomes and their RNA cargo were immunoprecipitated from fly head lysates as previously described (Huang et al., 2013). RNA was extracted from two independent TRAP samples, sequencing libraries generated and RNA-seq analyses carried out as described in Section Materials and Methods. Libraries were also generated for two independent total RNA samples isolated from the same head lysates used for TRAP. The raw sequence data (fastq files) have been submitted to NCBI SRA (submission ID SUB2126104; Bioproject PRJNA354880; accession numbers SRR5052811, SRR5052812, SRR5052813, and SRR5052814).

Based on the use of the STAR algorithm, more than 9000 genes were detected in the TRAP and total RNA samples (Table S1). Figure 1E shows the correlation between FPKM values derived from sequencing of the TRAP and total RNA libraries. FPKM values were correlated with a coefficient (adjusted r-squared) of 0.79, suggesting that expression values for most genes were similar in TRAP vs. total RNA. However, 1236 genes showed enriched expression in adult fly astrocytes (an IP/total FPKM ratio > 2, *q* ≤ 0.05; Figure 1F, Table S2). We verified enrichment for a group of genes with a range of FPKM values using qRT-PCR (Table S3). For the 14 assayed genes, astrocyte-enriched expression was observed with RNA-seq or qRT-PCR analysis (Figure S1), although absolute enrichment values differed between the two methods. Nonetheless, by either method, all of these genes showed enriched expression in astrocytes.

Brief descriptions of astrocyte-enriched gene functions were provided in an earlier publication that compared larval and adult fly astrocyte expression profiles (Huang et al., 2015). As noted in that publication, there is considerable evolutionary conservation for astrocyte-enriched fly genes. According to a recent version of FlyBase (FB2015_05, Nov. 2015; Attrill et al., 2016), 823 (~70%) of the 1236 adult-enriched genes have mammalian orthologs, and a large percentage of the mouse orthologs are known to be expressed in glial astrocytes. With reference to a recent RNA-seq dataset representing mouse glial subtypes (Zhang et al., 2014), 504/823 of the mouse orthologs (61%) show evidence of expression in astrocytes. As noted in the next section, the expression profiles of fly astrocytes and fly surface glia are quite different, consistent with the notion that the former class represents the counterpart of mammalian astrocytes.

**Figure 2:**
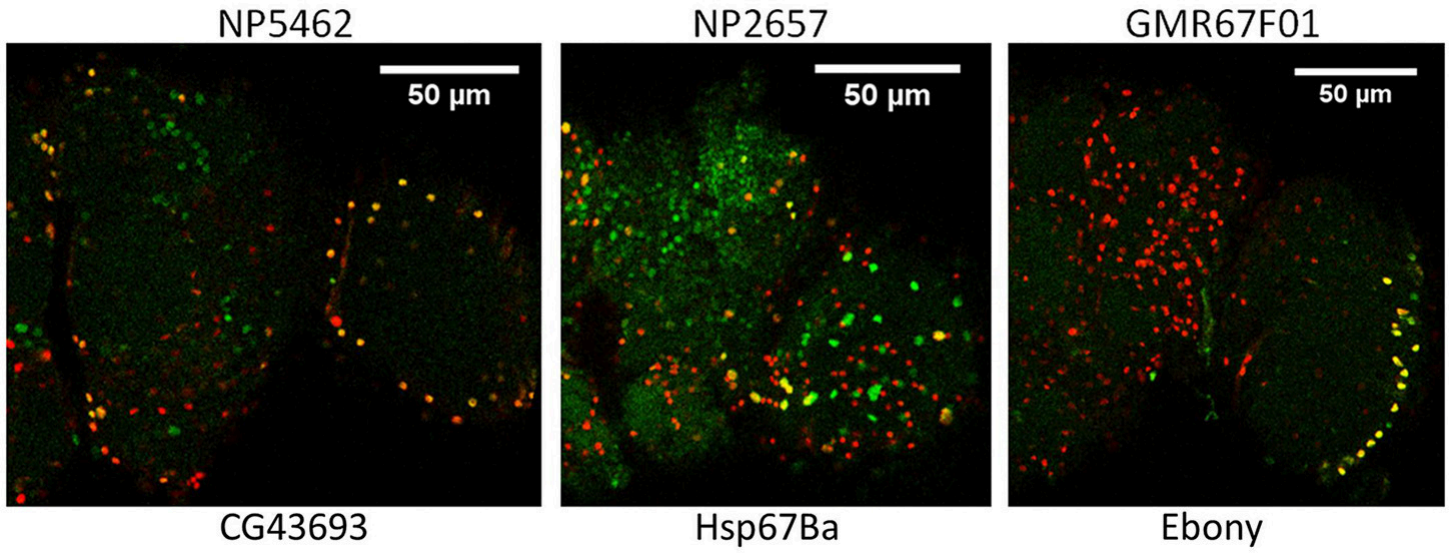
**Glial expression of Gal4 insertions mapping in or near 3 different genes that show astrocyte-enriched expression**. Red, Repo signal; green, GFP signal; yellow, overlap of repo and GFP signals. CG43693, Ebony and Hsp67Ba encode a putative amino acid transporter, β-alanyl amino acid synthase (BAS) and a protein chaperone, respectively. Gal4 insertions are upstream of Hsp67Ba and within Ebony and CG43693 (see FlyBase).

To verify glial expression for selected genes, we obtained 17 genomic insertions of Gal4 that were close to or within genes showing astrocyte-enriched expression from the Bloomington and National Institute of Genetics (NIG) stock collections. Nine different Gal4 insertions were associated with fluorescence when combined with UAS-GFP, and 5 of them exhibited expression in a subset of REPO-positive glial cells, consistent with expression in fly astrocytes. Figure 2 illustrates adult expression patterns for 3 different Gal4 insertions in or near CG43693 (a putative amino acid transporter), Hsp67Ba (a chaperone), and Ebony (β-alanyl-amine synthase). Of interest, the GMR67F01 Gal4 is inserted in or near Ebony, which has a glial-specific pattern of expression and is known to be required for normal rhythmicity (Suh and Jackson, 2007). Whereas Ebony is expressed in hundreds of adult glial cells, GMR67F01 driver expression is restricted to glial cells of the fly optic lobe. Identification of such region-specific drivers may facilitate characterization of glial cell subtypes that regulate behavior.

### Gene ontology (GO) analyses

**Figure 3:**
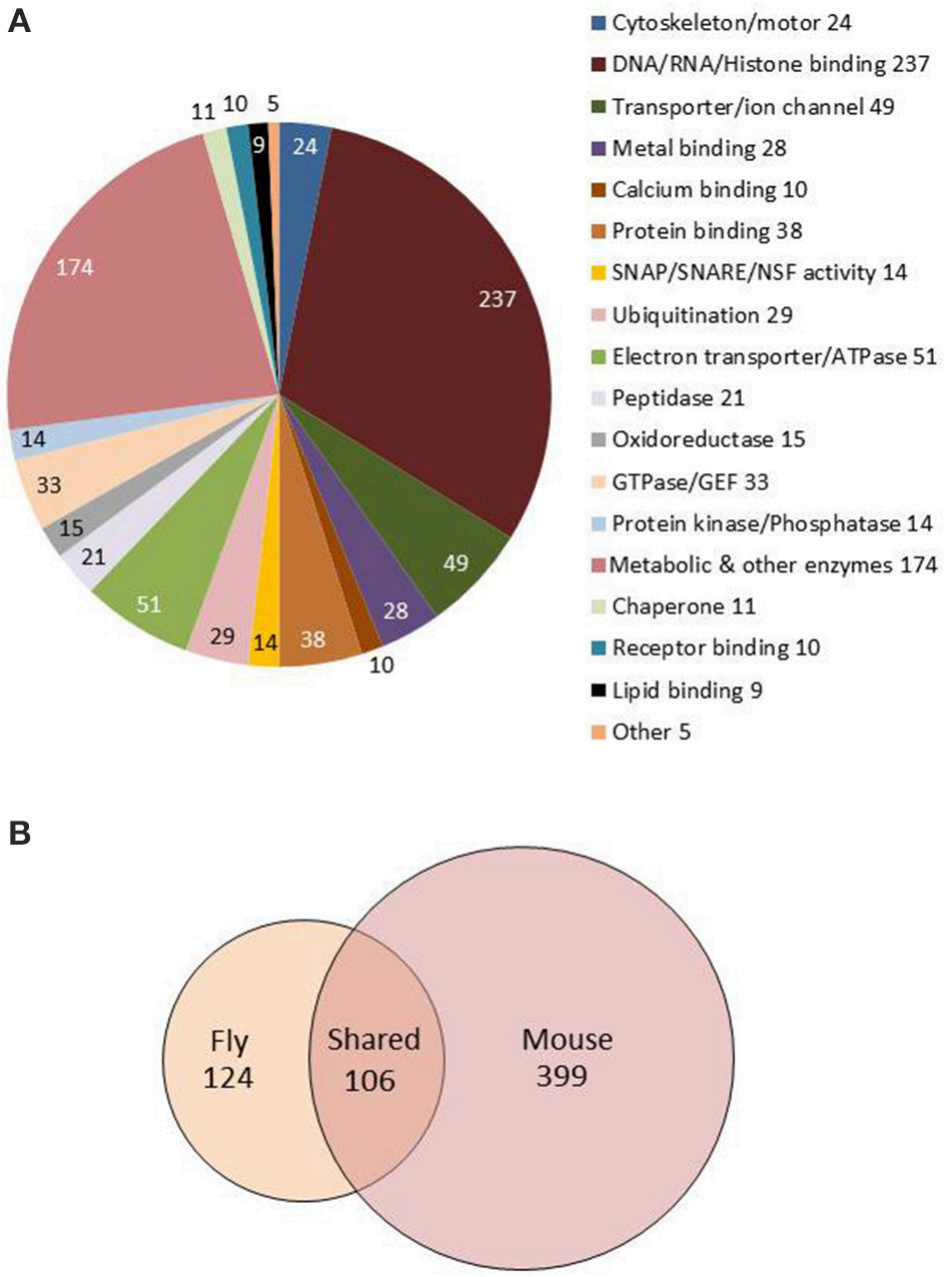
**Molecular functions and GO biological processes are conserved between fly and mouse astrocytes. (A)** Pie chart showing molecular functions for the 1236 genes showing astrocyte-enriched expression. Gene annotations were downloaded from FlyBase and manually sorted to derive this figure. **(B)** Venn diagram showing overlap of fly and mouse GO terms from the analysis of fly and mouse astrocyte-enriched genes (also see Table S4). One hundred six GO terms are conserved between the species (see text).

Version FB2015_05 of FlyBase contains annotations about molecular function for 919 of the adult-enriched genes, and this is summarized in Figure 3. Our analyses indicate that there are 49 genes with astrocyte-enriched expression encoding membrane transporters or ion channels, including Irk channels, the GABA transporter, an SLC5A transporter (CG9657) and GABA Transaminase (*GABAT*), a mitochondrial protein known to be expressed in glial cells and important for sleep regulation (Chen et al., 2015). Enriched or high-level expression was observed for other known fly glial genes in our analysis (Table S5): these encode Glutamine synthase 2, Glutamate oxaloacetate transaminase isoforms (*Got 1* and *Got2*), Dopamine acetyltransferase and Pyruvate decarboxylase (recently implicated in age-dependent memory impairment, Yamazaki et al., 2014). The glutamate transporter (*eaat1*) gene is expressed in fly astrocytes (~800 reads) but was not enriched relative to total RNA in our analysis (~1300 reads).

Fly genes showing astrocyte-enriched expression encode proteins associated with many significantly overrepresented biological processes (GO_BPs; Table S4). Overrepresented GO categories, determined using DAVID (Huang and Lempicki, 2009; see Section Materials and Methods) include metabolism and cellular energy (ATP) production, consistent with the neuronal support functions of fly and mammalian astrocytes (Brown and Ransom, 2007; Volkenhoff et al., 2015). In addition, gene expression, RNA splicing, protein degradation, oxidation/reduction, vesicle-mediated transport and secretion were overrepresented categories (Table S4, Sheet 1). The latter category includes syntaxins, sec homologs, NSF, SNAP, Aplip1, Amphiphysin, and Adaptor Protein Complex 1 and 2 subunits (AP-1 and AP-2), suggesting an important role for vesicle trafficking and secretion. Interestingly, some of the fly genes exhibit enriched expression in adult but not larval astrocytes (Huang et al., 2015). A similar analysis using mouse astrocyte-enriched genes (Table S4, Sheet 2; Zhang et al., 2014) showed that all of these categories (and more) were conserved between flies and mice (Table S4, Sheet 3, gray shading), highlighting the conservation of astrocyte function in the two species.

Using FlyBase annotations, we identified genes that may function in glia-neuron communication. We included those genes showing astrocyte-enriched expression (the set of 1236) and those with >100 reads in our dataset. For example, the fly homolog of Thrombospondin (Tsp), a secreted factor, is expressed at high levels in astrocytes, but does not show enriched expression in comparison to total head RNA. In addition, there are 60 astrocyte-expressed genes in the dataset that encode immunoglobulin (Ig) domain-containing proteins which may serve as glia-neuron signaling molecules. In addition, there are 118 astrocyte-expressed genes encoding small proteins with signal peptides, excluding odorant-binding and known cuticle proteins. With the exception of 3 of these proteins, all are <200 amino acids (aa) in length and 48 are <110 aa; they include factors such as Drosophila insulin-like peptides 2 and 6 (Dilp2 and 6), the latter known to be secreted from glial cells (Okamoto and Nishimura, 2015), Drosophila immune-induced proteins (DIMs) (Uttenweiler-Joseph et al., 1998; Clemmons et al., 2015), cytokine- and TNFα-like factors and others. Many of these proteins likely function as glia-neuron signaling molecules. We have examined flies expressing RNA interference transgenes representing at least 79 of the 118 genes, and several strains are associated with abnormal behavior (see later section).

We wondered how similar expression profiles were in different fly glial cell classes. A recent study derived the transcriptome of *Drosophila* surface glia (DeSalvo et al., 2014), an important glial cell class that forms the fly blood-brain barrier. Unlike our studies, that analysis employed fluorescence activated cell sorting (FACS) procedures and gene microarrays as a readout; it identified 2733 genes that showed ≥1.5-fold enriched expression in surface glia (SGCs) relative to brain total RNA. This analysis is an interesting complement to our studies as SGCs and astrocytes are predicted to have significant functional differences. Indeed, a comparison of astrocyte (the 1236 gene set) and the SG/brain enriched datasets identified only 135 genes in common (Table S6). Interestingly, these do not include any of the transporters (e.g., *nrv2, GAT*, CG9657, and *Ncc69*), secreted proteins (e.g., CG14141, CG1537, and CG1552) or other glial factors (e.g., *GABAT, AP2*-σ) that we (see below) and others have shown to be important for behavioral functions. Such divergent gene expression patterns underscore the distinct functions of fly astrocytes and surface glial cells.

### Astrocyte-expressed genes that regulate behavior

#### Effects on average activity level

Using the list of astrocyte-expressed genes, we initiated RNA interference (RNAi)-based genetic screens to identify factors important for glia-neuron signaling and adult behavior. These screens utilized the Gal4/UAS binary system and emphasized effects on locomotor activity, circadian behavior or bang sensitivity (an indicator of neuronal excitability). We chose to use the pan-glial *repo-Gal4* driver in this genetic screen, because of its strong expression in glia of the adult brain. In contrast, astrocyte-specific drivers often have weaker expression in certain brain regions. Thus, our RNAi-based screen potentially included classes of glia not represented in the TRAP-seq study.

In total, we screened 653 UAS-RNAi (UAS.IR) lines representing 318 genes (Table S7) encoding many different molecular functions: neuropeptide, peptidase, transporter/ion channel, calcium binding, DNA/RNA binding, and metabolism (37 of these genes are required for vesicle trafficking/release, and circadian phenotypes for several are described in a previous publication, Ng and Jackson, 2015). In the present study, all other genes of Table S7 were screened for effects on activity level and circadian locomotor activity; for more than two thirds, we examined two or more independent RNAi transgenes. For many, we expressed RNAi conditionally in adults and examined behavior (column D, Table S7).

**Figure 4:**
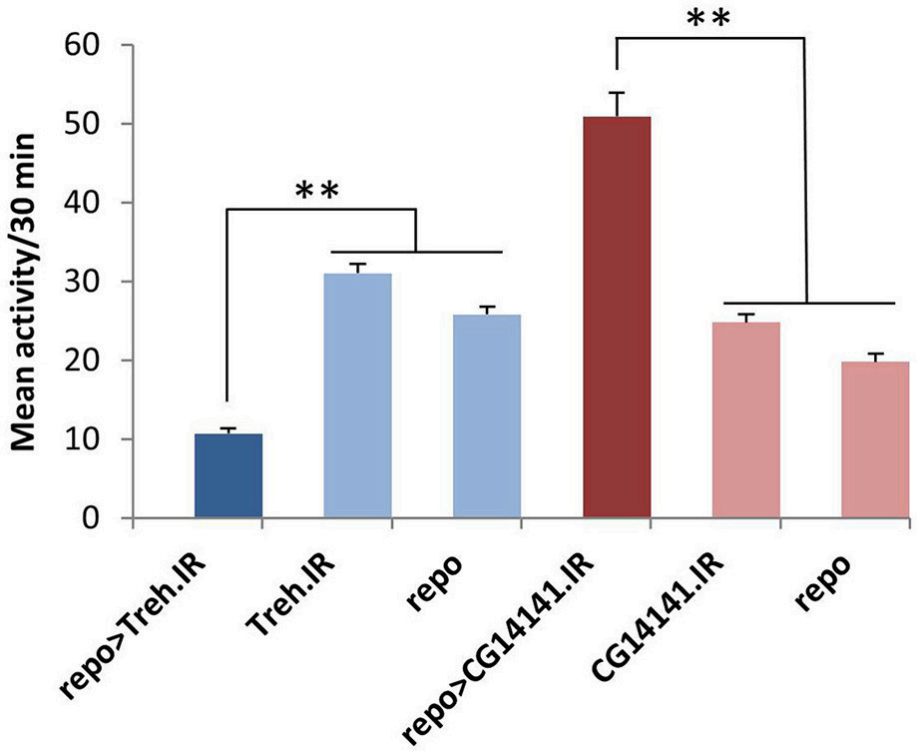
**Glial RNAi expression targeting several astrocyte-enriched genes resulted in altered activity level, 2 shown in this figure**. We used activity measures from across the diurnal cycle to calculate mean activity per 30 min period for RNAi-expressing populations (and controls). Glial expression of Treh.IR significantly decreased activity whereas expression of CG14141.IR increased activity level. ^**^p < 0.01.

Repo-Gal4-driven (pan-glial) expression of RNAi (IR) transgenes representing eight different genes was associated with significantly reduced or elevated locomotor activity in both light/dark (LD) and constant dark (DD) conditions with no effects on circadian period (Table 1). Examples of low and high activity strains are shown in Figure 4. We note that expression of RNAi targeting the gene (*eaat1*) encoding the major glial glutamate transporter resulted in lethality prior to the adult stage, and thus we could not examine behavior for such flies. Conditional adult expression of *eaat1* RNAi did not affect behavior, presumably because there was not a sufficient knockdown of the transporter. It was previously reported that larval and adult flies with GABA transporter (CG1732 or GAT) deficits have abnormal locomotor behavior (Stork et al., 2014), and we observed similar effects in adult flies (not shown).

Of note, five of the eight RNAi hits (*Treh, X16, CG1537, CG1552, and CG14141*) encode genes with significantly enriched fly astrocyte expression (≥1.5-fold) relative to total RNA from the lysate (see Section Materials and Methods). In addition, five of them have mammalian homologs (*Treh, Tsp, x16, 4EHP*, and *Spase25*) known to be expressed in astrocytes (Zhang et al., 2014), and four encode potentially secreted proteins that are likely to have glia-neuron signaling functions (*Tsp, CG1537, CG1552*, and *CG14141*; see Section Discussion). With the exception of *Treh* and *x16*, mutants are not available for these genes; therefore, we examined independent RNAi transgenes for as many as possible to exclude off-target effects. For *Treh, Tsp, 4EHP, CG1552*, and *CG1537*, independent RNAi transgenes had similar effects on activity level.

#### Genes associated with bang-induced paralysis

We screened a subset of genes (62; column C, Table S7) for effects of UAS-RNAi expression on bang sensitivity and adult locomotion using the *repo-Gal4* driver. These genes encode factors implicated in seizure susceptibility such as transporters, receptors, ion channels, calcium regulators, vesicle trafficking molecules and enzymes that control potential neurotransmitters levels (e.g., GABA, glutamate and dopamine). It has been shown, for example, that knockdown or mutation of glial exchangers (transporters) reduces the threshold for seizure-like activity (Melom and Littleton, 2013; Rusan et al., 2014). Indeed, pan-glial expression of RNAi transgenes targeting three different genes (*nrv2*, CG9657, and *Oat*) resulted in bang sensitivity and/or debilitated locomotor activity, both indicators of altered neuronal function. When *repo-Gal4*>*nrv2.IR* (VDRC 2660) flies were vortexed for 10 s (see Section Materials and Methods), they were rapidly paralyzed, exhibiting seizure-like activity upon recovery (Video S1). In three independent trials for *nrv2* (CG9261) carried out with biological replicates collected on three different days (each with 10 flies), an average of ~70% of flies became immobilized during a 45 s period of time, whereas control populations exhibited such behavior 0% (*repo-Gal4*) or 7% (*UAS-nrv2.IR*) of the time. Note that *nrv2.IR*- and CG9657.IR-expressing flies (see below) recovered from paralysis after several minutes (not shown); i.e., the effects were completely reversible.

Pan-glial expression of RNAi for another transporter gene, CG9657, also affected behavior. In three independent experiments, *repo-Gal4*>*CG9657.IR* (VDRC 43922) flies exhibited reversible paralysis behavior similar to that of *nrv2* RNAi flies (Video S2). In addition to the mechanical sensitivity, flies expressing CG9657.IR exhibited reduced activity and were completely arrhythmic (see next section). CG9657 is known to have a brain-specific expression pattern (Chintapalli et al., 2007); our TRAP results demonstrate that CG9657 has enriched expression in astrocytes relative to the total lysate, consistent with previous evidence indicating that the gene is expressed in fly glial cells (Freeman et al., 2003). Because CG9657 shows astrocyte-enriched expression, we also used a strong glia class-specific Gal4 driver (*eaat1-Gal4*) that expresses in most or all astrocytes and a few neurons of the optic lobe (Rival et al., 2004). To inhibit Gal4 activity in neurons, we included *elav-Gal80* in the genetic background. As with *repo-Gal4*>*CG9657.IR, eaat1-Gal4*>*CG9657.IR; elav-Gal80* flies exhibited a similar bang-induced paralysis (data not shown), consistent with an astrocyte requirement for the CG9657 gene.

Glial RNAi expression for a third gene (CG8782 or *Oat*) was associated with pupal lethality (VDRC 28952) or defective adult locomotion (VDRC 107178). In two independent experiments (one shown in Video S3), all *repo-Gal4*>*CG8782.IR* (VDRC 1071778) flies exhibited defective locomotion with some showing seizure-like activity after vials were gently banged. *Oat* is expressed at low levels in the adult brain according to FlyAtlas and our TRAP results are consistent with low expression in adult astrocytes (~300–400 reads in both astrocytes and the total lysate). *Oat* encodes mitochondrial ornithine aminotransferase, which converts ornithine to glutamic acid semialdehyde, a compound that may serve as a substrate for synthesis of glutamate and γ-amino butyric acid (GABA).

#### Effects on circadian behavior

**Figure 5:**
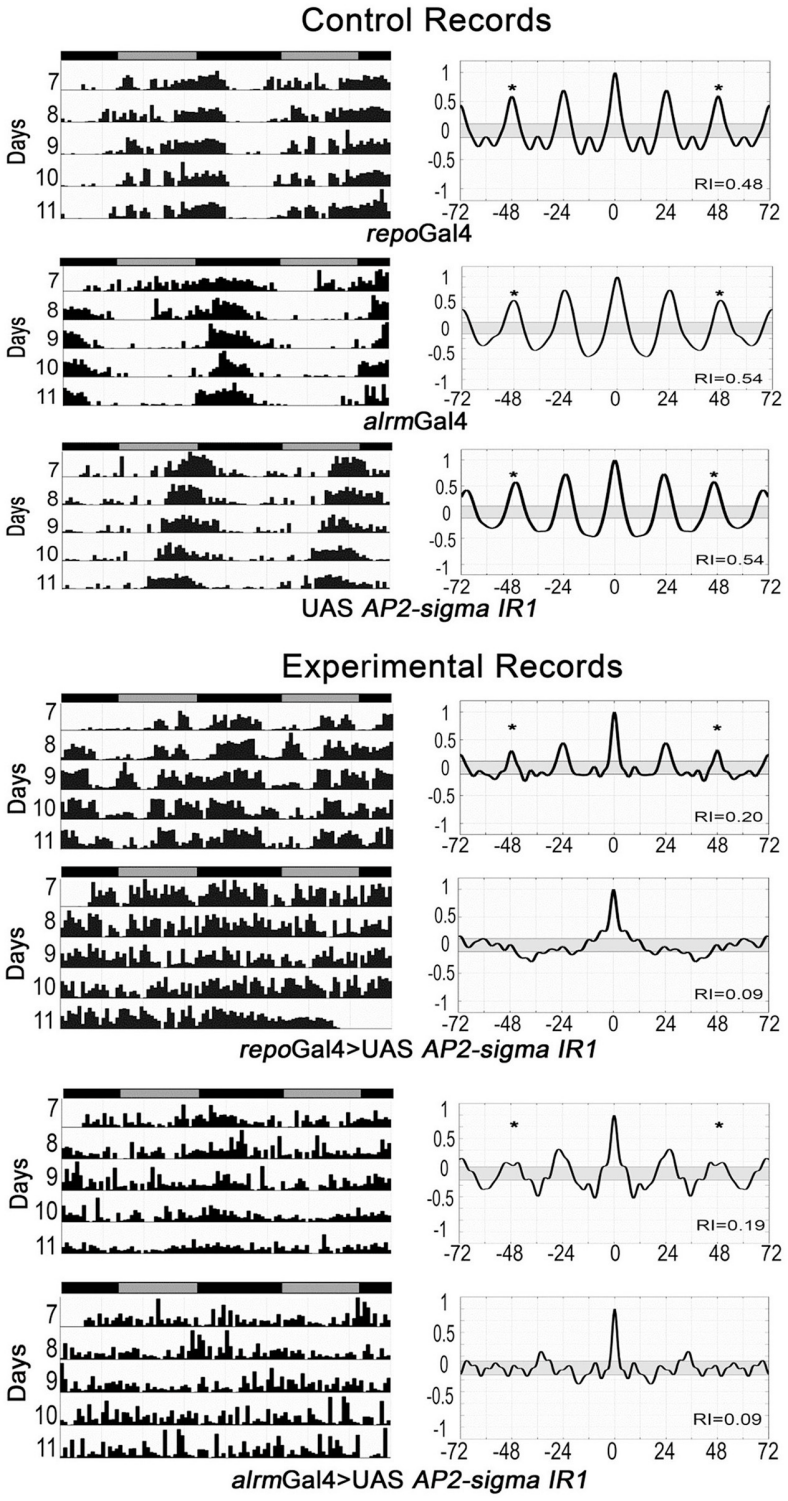
**Pan-glial or astrocyte-specific expression of AP2-σ RNAi caused behavioral arrhythmicity**. Representative actograms of locomotor activity in DD (left) and corresponding correlograms (right) are shown for both pan-glial (repo-Gal4) and astrocyte-specific (alrm-Gal4) AP2-σ RNAi expression along with their genetic controls. In this and subsequent figures, the third peak of the correlogram was used to calculate the Rhythmicity Index (RI) according to the Fly Toolbox package (Levine et al., 2002). The stars on correlograms indicate statistical significance, which was determined as *p* < 0.05. For each experimental population, (repo-Gal4>UAS AP2-σ. IR1 or alrm-Gal4>UAS AP2-σ. IR1), the upper panel shows a fly with weak rhythmicity and the lower panel shows an arrhythmic fly. Population data are included in Table 2.

Our previous studies have shown that glial factors modulate circadian behavior (Suh and Jackson, 2007; Ng et al., 2011) and that rhythmic behavior is a good readout for glia-neuron signaling (Ng et al., 2011; Ng and Jackson, 2015). Thus, in addition to examining activity level, we monitored circadian locomotor activity profiles of flies expressing RNAi transgenes for all genes listed in Table S7. A previous publication indicated that vesicle trafficking/release mechanisms are important for circadian behavior (Ng and Jackson 2015), consistent with the large number of small secreted proteins encoded by genes of our astrocyte expression dataset. Thus, we examined circadian behavior in flies expressing RNAi transgenes targeting an astrocyte-enriched vesicle recycling factor (AP2-σ). As shown in Figure 5, pan-glial deficits for AP2-σ resulted in altered circadian activity patterns; in this figure, actograms and associated correlograms are shown for control (top panels) and experimental (*repo-Gal4*>*AP2*-σ*.IR* (RNAi-expressing) flies (lower panels). Relative to control flies, those with pan-glial expression of a *UAS-AP2*-σ*.IR* transgene had less robust circadian rhythms than controls (uppermost *repo-Gal4*>*AP2*-σ*.IR* experimental record in Figure 5) or they were arrhythmic (lower *repo-Gal4*>*AP2*-σ*.IR* experimental record). Glial expression of either of two independent *UAS-AP2*-σ*.IR* transgenes (Table 2) affected the average Rhythmicity Index (RI), a statistical measure of robustness that is derived from correlogram analysis (Levine et al., 2002). Expression of either also led to a reduced percentage rhythmicity for the population (Table 2). Although, we did not examine a strong astrocyte class driver (e.g., *eaat1-Gal4*) with *UAS-AP2*-σ*.IR*, we did employ *alrm-Gal4*, a weaker Gal4 driver. Indicative of a requirement for the gene in astrocytes, *alrm-Gal4*>*UAS- AP2*-σ*.IR* populations had reduced RI and increased arrhythmicity (Figure 5, Table 2).

**Table 2:** **Effects on activity and/or rhythmicity index**.

**Strain**	**Gene**	**n**	**Activity ± SEM^α^**	**RI ± SEM^β^**	**Percent Rhythmic**	**Human Homolog**
*repo-Gal4>UAS-30001*	*Ncc69*^δ^^,^ ^γ^	19	27.4 ± 1.7	0.28 ± 0.03^***^	89	*Nkcc1-2*^π^
*UAS-30001*		21	33.8 ± 2.0	0.55 ± 0.02	100	
*repo-Gal4*		25	27.8 ± 1.9	0.55 ± 0.02	100	
*repoGal4>UAS-28682*	*Ncc69*	25	20.8 ± 1.4	0.36 ± 0.02^***^	96	*Nkcc1-2*
*UAS-28682*		28	27.7 ± 3.6	0.57± 0.02	100	
*repo-Gal4*		31	28.9 ± 1.8	0.59 ± 0.02	100	
*repo-Gal4>UAS-104942*	*Npc2g*^δ^^,^ ^γ^	47	12.4 ± 0.5^**^	0.34 ± 0.02^**^	87	*Npc2*
*UAS-104942*		64	22.6 ± 0.3	0.54 ± 0.01	100	
*repo-Gal4*		42	21.7 ± 0.8	0.48 ± 0.01	100	
repo-Gal4>UAS-11314R-2	*Npc2g*	8	30.5 ± 3.3	0.35 ± 0.10^***^	**75**	*Npc2*
UAS-11314R-2		8	32.6 ± 2.4	0.63 ± 0.07	100	
*repo-Gal4*		15	25.9 ± 2.2	0.58 ± 0.02	100	
*repoGal4>UAS-50721*	*Npc2g*	8	15.5 ± 1.2^**^	0.35 ± 0.09^*^	**50**	*Npc2*
*UAS-50721*		8	27.4 ± 3.0	0.57 ± 0.07	100	
*repo-Gal4*		15	25.9 ± 2.2	0.58 ± 0.02	100	
*repo-Gal4>UAS-27322*	*AP-2*σ^ϵ^^,^ ^γ^	24	19.0 ± 1.5	0.24 ± 0.03^**^	**64**	*AP-1 & 2*
*UAS-27322*		28	24.4 ± 1.8	0.42 ± 0.02	100	
*repo-Gal4*		27	24.9 ± 1.4	0.41 ± 0.02	100	
*repo-Gal4>UAS-34148*	*AP-2σ*	15	10.3 ± 1.4^**^	0.17 ± 0.03^*^	**67**	*AP-1 & 2*
*UAS-34148*		24	22.0 ± 1.9	0.39 ± 0.02	92	
*repo-Gal4*		27	19.0 ± 1.1	0.27 ± 0.02	89	
*alrm-Gal4>UAS-27322*	*AP-2σ*	16	28.5 ± 1.9	0.29 ± 0.04^*^	**69**	*AP-1 & 2*
*UAS-27322*		16	21.8 ± 2.2	0.43 ± 0.04	100	
*alrm-Gal4*		12	26.4 ± 2.2	0.55 ± 0.02	100	
*repo-Gal4>UAS-43922*	*CG9657*^ϵ^^,^ ^ϕ^^,^ ^γ^	13	4.0 ± 0.3^***^	0.11 ± 0.02^***^^χ^	**0**	*Slc5A*^μ^
*UAS-43922*		16	31.1 ± 2.8	0.61 ± 0.02	100	
*repo-Gal4*		16	15.0 ± 1.2	0.45 ± 0.04	100	

α *Average activity/30 min in LD*.

β *Rhythmicity Index, calculated from DD data*.

χ *Flies did not synchronize well to LD*.

δ *Astrocyte expression is approximately the same as that observed in the total lysate*.

ϵ *Astrocyte-enriched expression*.

ϕ *Nervous system-specific expression*.

γ *Mouse ortholog has astrocyte expression*.

μ *Sodium-coupled Moncarboxylate Transporter 1–2*.

π *SLC12A Na^+^/K^+^/Cl^+^ transporter. ^π^Only 1 RNAi line exists for this gene*.

* *p < 0.05*;

** *p < 0.01*;

*** *p < 0.001*.

*Bold values indicate experimental genotypes that differ significantly from controls*.

A knockdown of CG11314, encoding a Niemann-Pick type C-2 (NPC2) protein, resulted in reduced activity and/or decreased RI for 3 independent RNAi constructs targeting this gene (Table 2). In addition, the expression of 2 of these constructs decreased percent rhythmicity (>25% decreased) relative to controls; for one construct, only 50% of flies exhibited significant rhythmicity. CG11314 corresponds to *Npc2g*, one of eight *Npc2* genes encoding fly NPC2 proteins (Fluegel et al., 2006). These fly proteins are important for cholesterol transport and steroid hormone synthesis (see Section Discussion).

**Figure 6:**
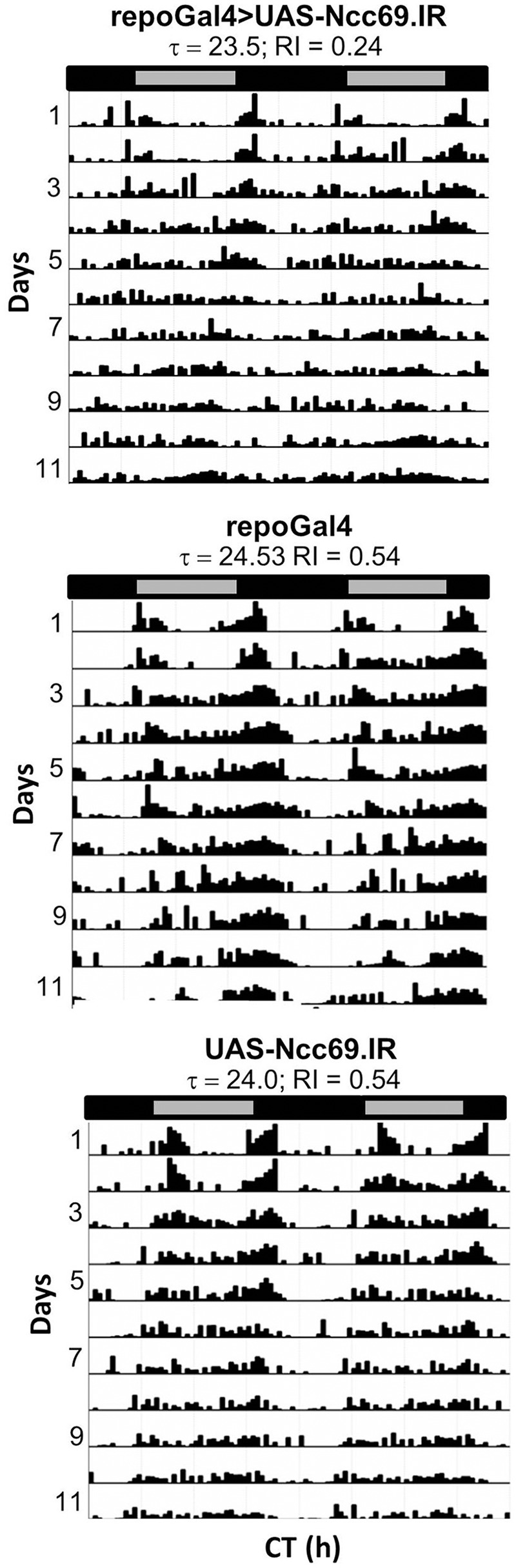
**Pan-glial expression of Ncc69.IR resulted in decreased rhythmicity**. Representative actograms of flies with pan-glial expression of Ncc69 RNAi and controls. Data were collected in DD. RI values and circadian periods were calculated using Fly Toolbox. Population data are included in Table 2.

**Figure 7:**
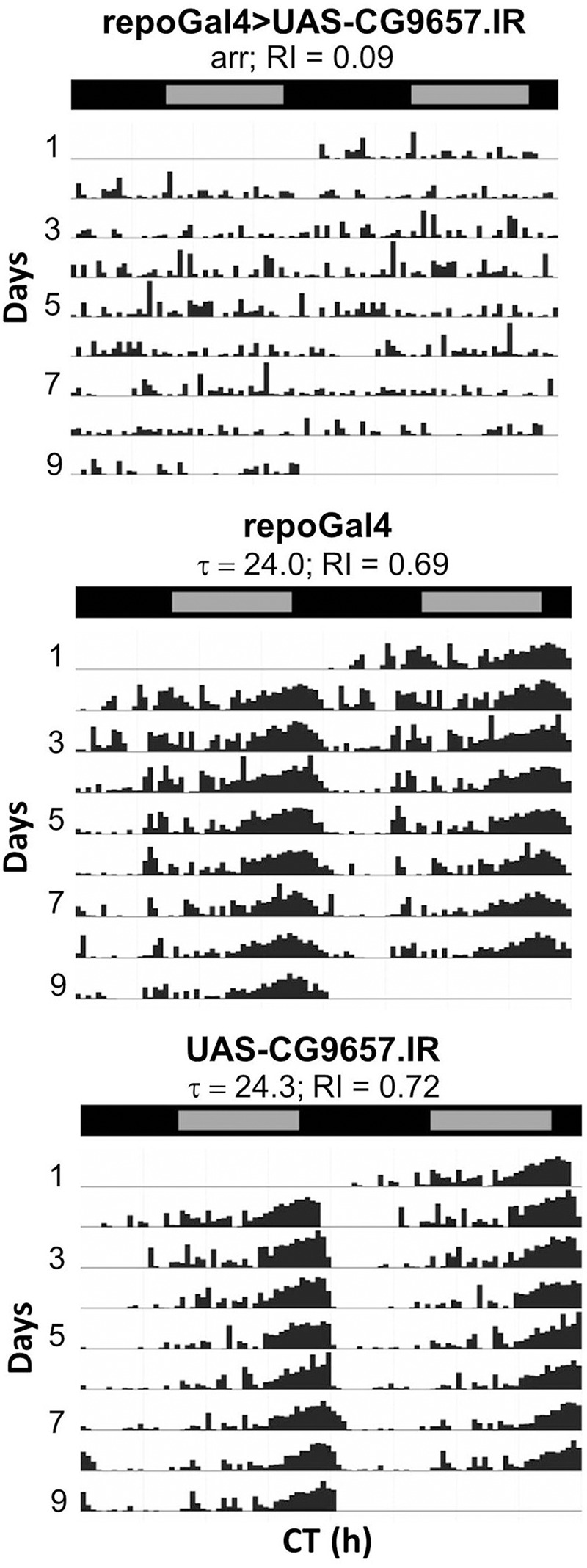
**Pan-glial expression of CG9657.IR caused arrhythmic locomotor activity**. Representative actograms for control flies or those expressing CG9657.IR. Data were collected in DD. Population data are included in Table 2.

RNAi transgenes targeting two different glial transporter genes (*Ncc69* and CG9657) were associated with altered rhythmicity, one of which (CG9657) exhibits astrocyte-enriched expression. Whereas RNAi transgenes targeting *Ncc69* reduced the average RI (i.e., weakened rhythms) with little effect on percent rhythmicity (Table 2; Figure 6), a transgene targeting CG9657 had a more severe effect on activity level, RI and percent rhythmicity (Table 2; Figure 7). Although repo-Gal4>CG9657.IR flies had reduced activity and exhibited bang-induced paralysis (see previous section), they did not appear to be locomotor defective (i.e., they walked normally). Nonetheless, activity was completely aperiodic in all flies examined in two independent experiments (one shown in Table 2). Similarly, *eaat1-Gal4*>*CG9657.IR; elav-Gal80 flies*, which express the RNAi more selectively in astrocytes, were less active and arrhythmic (data not shown), consistent with a requirement in astrocytes. Of interest, the vast majority of RNAi-expressing flies (using either *repo-Gal4* or *eaat1-Gal4*) failed to entrain to the LD cycle (data not shown), indicative of a general problem with the circadian circuit. Importantly, we do not think that the reduced activity of CG9657.IR-expressing flies causes apparent arrhythmic behavior as other strains with low activity had normal circadian behavior (Table 1). Although only 1 of 3 CG9657 RNAi transgenes affected behavior (not an uncommon finding in RNAi-based screens) the one causing circadian arrhythmicity is not predicted to have any genomic off targets.

Given the severe circadian phenotype of flies expressing CG9657 RNAi, we examined the nervous system to determine whether the circadian circuitry was compromised. We co-stained CG9657 RNAi flies and controls (UAS or Gal4 alone) with antibodies recognizing PERIOD (PER) clock protein and Pigment Dispersing Factor (PDF), the major clock neuronal transmitter. We found that PER-containing neurons were normal in position and number (Figure S2 and data not shown). Given the inherent variability in PDF projection morphology, we employed a blind scoring method (see Section Materials and Methods) to assess these measures. Brain hemispheres were coded by one person (AMY) and blind scoring was performed by 2 of us (FRJ and SY) with experience in visualizing the PDF cell bodies and projections. In one experiment, we observed significant morphological abnormality for the l-LNv optic lobe dendritic arbors and contralateral projections (within the POT) in CG9657 RNAi flies, relative to control flies. However, this phenotype was not observed in 3 additional biological replicates (two pooled in the results shown in Figure S3). Thus, we conclude that there are no significant morphological differences between the PDF circuits of CG9657.IR-expressing flies and controls.

## Discussion

In these and previous studies, we have described the molecular profile of the Drosophila astrocyte class of glial cells. With this database as a foundation, we initiated an RNAi-based screen to identify genes required for adult behavior. We describe 3 classes of interesting encoded factors in the following sections.

### Secreted proteins and factors relevant for secretion

RNAi expression for a number of genes with potential secretory functions resulted in reduced activity or circadian arrhythmicity (*AP-2*σ*; Spase25, Tsp*, and genes encoding small secreted proteins). AP2-σ is a component of the AP2 complex, which along with clathrin and other factors is required for clathrin-mediated endocytosis (McMahon and Boucrot, 2011). In neurons, this process is critical for neurotransmitter vesicle recycling. To our knowledge, a similar requirement for the clathrin complex has not been documented for astrocytes, although vesicular exocytosis occurs and recycling events can be visualized in these cells using imaging methods (Bezzi and Volterra, 2014a,b). As mentioned in a previous publication, fly astrocytes express conserved vesicle recycling and release factors (Huang et al., 2015). Consistent with the importance of glial secretion mechanisms, activity is reduced in flies expressing RNAi targeting *Spase25*; this gene encodes the Signal Peptidase Complex Subunit 2, required for signal peptide processing.

Several novel secreted proteins (CG1552, CG14141, and CG1537) are required for normal behavior. CG1552 encodes 171 and 184 amino acid (aa) proteins with N-terminal hydrophobic sequences that are predicted to serve as signal peptides (SignalP 4.0; Petersen et al., 2011); the gene has a brain-specific pattern of expression according to FlyAtlas (Chintapalli et al., 2007). CG14141 encodes a 162-aa Ig-domain protein—expressed only in the brain and eye of adults—that is almost certainly a secreted factor based on SignalP analysis and the known function of a *C. elegans* ortholog (Rapti et al., 2011). CG1537 encodes a 135-aa protein of unknown function that is also likely to be secreted (with a cutoff of 0.45 using SignalP).

Interestingly, both CG1537 and CG14141 have fly homologs that reside in adjacent genomic intervals, indicative of gene duplication events. CG14141 is 50% identical to CG7607 another astrocyte-enriched gene that encodes an Ig domain protein with nervous system-specific expression; however, RNAi targeting CG7607 did not produce a behavioral phenotype. CG1537 expresses a 135-aa protein with moderate brain expression and little expression in other adult tissues. It has homology to the adjacent gene CG1545; however, we have not examined flies expressing RNAi that targets CG1545.

Flies expressing RNAi targeting the conserved *Tsp* gene also have reduced activity. This gene encodes Thrombospondin, a factor known to be secreted from mammalian astrocytes and important for synaptogenesis (Christopherson et al., 2005) as well as adult responses to injury (TSP and its receptor α2δ-1 are upregulated with cortical injury, Andresen et al., 2014). Of relevance, a putative fly TSP receptor (Straightjacket), with 45% similarity to mouse α2δ-1, is expressed at a low level in both astrocytes and neurons according to our study (Table S1) and published results (Thomas et al., 2012). Thus, fly TSP may serve as a ligand for α2δ-1, similar to mammals and be relevant for glia-neuron signaling. Overall, our results underscore a role for glial secretion in the regulation of behavior, and identify several factors that are required for this function.

### Potential metabolic support proteins

Several genes associated with altered behavior may be relevant for the metabolic support of neurons (*Treh, Npc2g, CG9657*). A glial deficit for Treh, which catalyzes conversion of trehalose to glucose in both vertebrates and invertebrates, significantly reduces fly activity (Table 1). From recent studies, it is known that fly trehalose is metabolized predominantly in glial cells, rather than neurons. Indeed, glycolysis seems to be a glial rather than neuronal requirement in both flies and mammals, with glial metabolites (e.g., lactate) then being provided to neurons (Poitry-Yamate et al., 1995; Gordon et al., 2008; Rouach et al., 2008; Volkenhoff et al., 2015; Schirmeier et al., 2016). It has also been demonstrated that fly *Treh* is predominantly expressed in glial cells (Volkenhoff et al., 2015), consistent with our TRAP profiling results, suggesting enrichment of expression in these cells. Thus, altered behavior of Treh-deficient flies is consistent with the importance of this enzyme and other metabolic factors for the glial glycolytic support of fly neurons.

CG9657 encodes a SLC5A-type transporter, with a mammalian homolog that is known to have astrocyte-specific expression (SLC5A12; Ganapathy et al., 2008). According to HUGO Gene Nomenclature (Seal et al., 2011), vertebrate SLC5A homologs transport moncarboxylates (e.g., pyruvate and lactate), idodide, choline or vitamins; thus, they may couple the transport of lactate—a downstream product of Treh activity—or another metabolite from glia to neurons. Given the importance of astrocyte-neuron metabolic coupling, CG9657 may be important for the metabolic support of neuronal function. Of interest, RNAi expression for either CG9657 or *Treh* reduces locomotor activity but only CG9657 is associated with circadian arrhythmicity. Thus, *Treh* and CG9657 deficits either affect glial and neuronal metabolism in distinct ways or the function of the transporter is more selective for the circadian circuitry. Interestingly, expression of CG9657 RNAi is associated with arrhythmicity; RNAi-expressing flies do not entrain well to LD and are arrhythmic in DD. However, we could not detect significant differences in clock neuron number/distribution or PDF circuitry. Therefore, the arrhythmic behavior may reflect alterations of clock output circuits other than those of the PDF neurons.

Multiple RNAi transgenes targeting *Npc2g*, which is expressed at high levels in fly astrocytes (Table S1), are associated with reduced activity and/or arrhythmic behavior (Table 2). In mammals, glial NPCs (NPC1 and NPC2 isoforms) mediate cholesterol transport—important for steroid synthesis and other functions—from late endosomes or lysosomes to the ER and plasma membrane. Together with apolipoprotein E (APOE)—a secreted cholesterol transporter—the NPCs are required for shuttling cholesterol from astrocytes to neurons in support of neuronal functions (Vance, 2012). Thus, it is notable that the *Apoltp* gene, with lipid transporter activity, is also expressed in fly astrocytes (Table S1). Of interest, fly NPCs are known to be important for the synthesis of ecdysteroid (20E), a hormone produced during development and in adult tissues (Fluegel et al., 2006; Huang et al., 2007). To our knowledge there is no evidence of local ecdysteroid synthesis in the fly nervous system, but receptors for the hormone (nuclear hormone receptors) are present in this tissue (Truman et al., 1994). Moreover, clock neurons themselves express ecdysteroid-responsive nuclear hormone receptors (e.g., UNF and E75), as well as a membrane ABC transporter (E23) postulated to regulate hormone responsiveness, and these are required for normal circadian rhythmicity (Itoh et al., 2011; Kumar et al., 2014; Jaumouillè et al., 2015). Evidence suggests that these factors serve as components of the molecular clock in Drosophila and more primitive insects (Kamae et al., 2014); they may respond to hormone to regulate specific genes, the molecular clock and behavior.

Ecdysteroid can be detected in adult fly heads (Ishimoto and Kitamoto, 2010), and in addition to circadian behavior it regulates both sleep and memory processes (Ishimoto and Kitamoto, 2011). Our profiling results did not detect expression of genes encoding steroid (ecdysteroid) synthetic enzymes (the Halloween family of genes) in astrocytes (Table S1). Thus, if glial NPCs contribute to local steroid synthesis in head tissues, other cell types must be involved. Perhaps fly astrocytes provide cholesterol to adipose tissue of the head (the fat body) which do express the Halloween genes (T. Kitamoto, pers comm.). Interactions between the fat body and behavior have previously been documented (Lazareva et al., 2007).

### Glial transporters

Glial deficits for CG9657 and nrv2 (this paper) as well as Ncc69 (Rusan et al., 2014) are associated with enhanced seizure susceptibility. CG9657 is predicted to be a metabolite transporter (see above), whereas Nrv2 is the non-catalytic β subunit of a brain transmembrane Na+/K+ transporter (sodium pump); it regulates the number of catalytically active sodium pumps at the plasma membrane and thus the membrane electrochemical gradient. Nrv2 is known to be expressed in glial cells and has very high expression in the adult brain (FlyAtlas); our TRAP results indicate that expression in astrocytes is equivalent to that observed in the total lysate (~2000 reads). Nrv2 protein shows diurnal rhythms in abundance when examined in whole head preparations (Gorska-Andrzejak et al., 2009). Thus, it may be required for the diurnal regulation of the glial electrochemical gradient.

The Ncc69 transporter is the fly ortholog of vertebrate NKCC1 and 2, SLC12A family members that are important for sodium, potassium and chloride transport and maintenance of ionic balance in the nervous system. Knockdown of Ncc69 in glia may result in altered ionic balance and neuronal function. Of interest, a previous study showed that RNAi-based knockdown of *Ncc69* in glial cells, but not neurons, caused seizure susceptibility and swelling of peripheral nerves in larval *Drosophila* (Rusan et al., 2014). Although we did not examine seizure susceptibility in *Ncc69* knockdown flies, our studies show that such flies have weakened circadian activity rhythms (Table 2). Of related interest, a previous expression profiling study from our lab suggests that *Ncc69* mRNA exhibits circadian changes in abundance (Huang et al., 2013), and perhaps this rhythm is important for normal circadian activity. In contrast to *Ncc69*, we did not observe effects on behavioral rhythms in flies expressing *nrv2* RNAi. However, it is known that the alpha and beta subunits of fly Na+/K+ ATPase exhibit circadian changes in abundance within epithelial glia and other cells of the optic neuropil (Gorska-Andrzejak et al., 2009). These studies of *Ncc69* and *nrv2* suggest that there may be circadian regulation of ion transport within glia and other cell types.

## Conclusions

Results from this study are consistent with a requirement for glia-neuron communication, by direct signaling or metabolic coupling, in the regulation of adult behavior. Our TRAP analysis shows a striking similarity in the expression profiles of fly and mammalian astrocytes, indicative of evolutionary conservation; in contrast, fly astrocyte and surface glia have distinct expression profiles. Consistent with conservation, more than half of the genes identified in the reported RNAi-based genetic screen are known to have vertebrate homologs, with many of them exhibiting astrocyte expression in flies and mammals. Our results lay the foundation for further studies of glia-neuron signaling in the context of behavior. Given the evolving tools for genetic analysis in the mouse system, i.e., CRISPR/Cas9-based cell-specific gene manipulation, it will be possible to assess the functions of conserved genes in mammalian behavior.

## Author contributions

FN, SS directed and conducted genetic screens and analyzed behavioral data; YH performed and analyzed TRAP profiling data and did GO analyses; AY performed immunostaining analyses; SY participated in genetic screens and analyzed behavioral data; MR participated in genetic screens and analyzed behavioral data; LI helped with analysis of TRAP profiling data; YY helped with analysis of TRAP profiling results; FJ directed the research and wrote the manuscript.

### Conflict of interest statement

The authors declare that the research was conducted in the absence of any commercial or financial relationships that could be construed as a potential conflict of interest.

## Supplementary material

Figure S1 **Fourteen genes with various FPKM values show astrocyte enrichment (TRAP compared to total RNA) by either RNA-seq or QPCR analysis**. QPCR methods are described in the Section Materials and Methods. In the case of most genes showing greater than ~8-fold enrichment by RNA-seq (6 out of 7), a similar or higher enrichment value was observed with QPCR. In contrast, those genes showing lower enrichment by RNA-seq showed reduced enrichment when assayed by QPCR.

Figure S2 **Knockdown of CG9657 did not affect the number of PER-containing cells. (A)** control brain hemisphere, **(B)** *repo-Gal4*>*CG9657.IR* hemisphere. DN1, Dorsal neurons 1; DN3, Dorsal neurons 3; LNv, ventral lateral neurons; LND, Dorsal lateral neurons. Note the different scales for the hemispheres shown in **(A,B)**. More than 30 brain hemispheres were examined for each genotype.

Figure S3 **Quantification of PDF projection morphology in control brains and those with pan-glial expression of CG9657.IR (see Section Materials and Methods for details)**. In three biological replicates (two pooled in this figure), glial expression of CG9657.IR did not result in abnormal morphology for s-LNv dorsal projections (top), l-LNv contralateral (POT) projections (middle) or l-LNv optic lobe projections (bottom) in comparison to both UAS and GAL4 genetic controls. While there was altered morphology for optic lobe projections in CG9657.IR-expressing flies compared to the UAS control, there was no significant difference between experimental and Gal4 control brains. Results are mean ± SEM; *n* = 23–30 brain hemispheres; ^*^*p* < 0.05 for a one-way ANOVA with Tukey's multiple comparison test; ns, not significant.

Table S1 **Raw counts of genes detected by TRAP in fly astrocytes or in total RNA from head lysates. Two biological replicates are shown for each type of sample**.

Table S2 **Genes with enriched astrocyte expression detected by TRAP analysis**.

Table S3 **qRT-PCR verification of enrichment for selected astrocyte genes**.

Table S4 **Biological processes that are significantly overrepresented in fly and mouse genes with astrocyte-enriched expression**. Biological processes (BPs) were associated with 932 of the astrocyte-enriched fly genes (this tab one) and 1252 mouse genes (tab 2). *P*-values were calculated using Fisher's. Shows GO_BP terms that are conserved between fly and mouse astrocyte-enriched genes. The mouse genes are from Zhang et al. (2014).

Table S5 **Genes detected by TRAP profiling that are known to be expressed in fly glia**.

Table S6 **Genes showing enriched expression in both surface glia and astrocytes**.

Table S7 **All genes screened for circadian phenotypes by pan-glial RNAi expression (column A)**. For selected genes (column D), we expressed RNAi transgenes conditionally in adults and examined circadian behavior. For a subset of genes (column C), we tested RNAi-expressing flies for bang sensitivity.

Video S1 **Pan-glial RNAi expression for *nrv2* causes bang-induced paralysis**. Vial 1, *repo-Gal4*>*nrv2.IR*; vial 2, *UAS-nrv.IR*; vial 3, *repo-Gal4*.

Video S2 **Pan-glial RNAi expression for CG9657 causes bang-induced paralysis**. Vial 1, *repo-Gal4*>*CG9657.IR*; vial 2, *UAS-CG9657.IR*.

Video S3 **Pan-glial RNAi expression for *Oat* causes bang-induced paralysis**. Vial 1, *repo-Gal4*; vial 2, *UAS-Oat.IR*; vial 3, *repo-Gal4*>*Oat.IR*.

## References

[B1] Allen N. J. Bennett M. L. Foo L. C. Wang G. X. Chakraborty C. Smith S. J.. (2012). Astrocyte glypicans 4 and 6 promote formation of excitatory synapses via GluA1 AMPA receptors. Nature 486, 410–414. 10.1038/nature11059 22722203 PMC3383085

[B2] Anders S. Pyl P. T. Huber W. (2014). HTSeq—a Python framework to work with high-throughput sequencing data. Bioinformatics 31, 166–169. 10.1093/bioinformatics/btu638 25260700 PMC4287950

[B3] Andresen L. Hampton D. Taylor A. Morel L. Yang Y. Maguire J.. (2014). Gabapentin attenuates hyperexcitability in the freeze-lesion model of developmental cortical malformation. Neurobiol. Dis. 71, 305–316. 10.1016/j.nbd.2014.08.022 25158291 PMC4179994

[B4] Araque A. Carmignoto G. Haydon P. G. Oliet S. H. Robitaille R. Volterra A. (2014). Gliotransmitters travel in time and space. Neuron 81, 728–739. 10.1016/j.neuron.2014.02.007 24559669 PMC4107238

[B5] Attrill H. Falls K. Goodman J. L. Millburn G. H. Antonazzo G. Rey A. J.. (2016). FlyBase: establishing a gene group resource for *Drosophila melanogaster*. Nucleic Acids Res. 44, D786–D792. 10.1093/nar/gkv1046 26467478 PMC4702782

[B6] Awasaki T. Lai S. L. Ito K. Lee T. (2008). Organization and postembryonic development of glial cells in the adult central brain of Drosophila. J. Neurosci. 28, 13742–13753. 10.1523/JNEUROSCI.4844-08.2008 19091965 PMC6671902

[B7] Bezzi P. Volterra A. (2014a). Identification and staining of distinct populations of secretory organelles in astrocytes. Cold Spring Harb. Protoc. 2014, 532–536. 10.1101/pdb.prot081703 24786508

[B8] Bezzi P. Volterra A. (2014b). Imaging exocytosis and recycling of synaptic-like microvesicles in astrocytes. Cold Spring Harb. Protoc. 2014, 537–543. 10.1101/pdb.prot081711 24786509

[B9] Borycz J. Borycz J. A. Edwards T. N. Boulianne G. L. Meinertzhagen I. A. (2012). The metabolism of histamine in the *Drosophila optic* lobe involves an ommatidial pathway: beta-alanine recycles through the retina. J. Exp. Biol. 215, 1399–1411. 10.1242/jeb.060699 22442379 PMC3309881

[B10] Brown A. M. Ransom B. R. (2007). Astrocyte glycogen and brain energy metabolism. Glia 55, 1263–1271. 10.1002/glia.20557 17659525

[B11] Chen W. F. Maguire S. Sowcik M. Luo W. Koh K. Sehgal A. (2015). A neuron-glia interaction involving GABA transaminase contributes to sleep loss in sleepless mutants. Mol. Psychiatry 20, 240–251. 10.1038/mp.2014.11 24637426 PMC4168011

[B12] Chintapalli V. R. Wang J. Dow J. A. (2007). Using FlyAtlas to identify better Drosophila melanogaster models of human disease. Nat. Genet. 39, 715–720. 10.1038/ng2049 17534367

[B13] Christopherson K. S. Ullian E. M. Stokes C. C. Mullowney C. E. Hell J. W. Agah A.. (2005). Thrombospondins are astrocyte-secreted proteins that promote CNS synaptogenesis. Cell 120, 421–433. 10.1016/j.cell.2004.12.020 15707899

[B14] Clarke L. E. Barres B. A. (2013). Emerging roles of astrocytes in neural circuit development. Nat. Rev. Neurosci. 14, 311–321. 10.1038/nrn3484 23595014 PMC4431630

[B15] Clemmons A. W. Lindsay S. A. Wasserman S. A. (2015). An Effector Peptide Family Required for Drosophila Toll-Mediated Immunity. PLoS Pathog. 11:e1004876. 10.1371/journal.ppat.1004876 25915418 PMC4411088

[B16] Danjo R. Kawasaki F. Ordway R. W. (2011). A tripartite synapse model in Drosophila. PLoS ONE 6:e17131. 10.1371/journal.pone.0017131 21359186 PMC3040228

[B17] DeSalvo M. K. Hindle S. J. Rusan Z. M. Orng S. Eddison M. Halliwill K.. (2014). The Drosophila surface glia transcriptome: evolutionary conserved blood-brain barrier processes. Front. Neurosci. 8:346. 10.3389/fnins.2014.00346 25426014 PMC4224204

[B18] Dobin A. Davis C. A. Schlesinger F. Drenkow J. Zaleski C. Jha S.. (2013). STAR: ultrafast universal RNA-seq aligner. Bioinformatics 29, 15–21. 10.1093/bioinformatics/bts635 23104886 PMC3530905

[B19] Doherty J. Logan M. A. Tasdemir O. E. Freeman M. R. (2009). Ensheathing glia function as phagocytes in the adult Drosophila brain. J. Neurosci. 29, 4768–4781. 10.1523/JNEUROSCI.5951-08.2009 19369546 PMC2674269

[B20] Fluegel M. L. Parker T. J. Pallanck L. J. (2006). Mutations of a Drosophila NPC1 gene confer sterol and ecdysone metabolic defects. Genetics 172, 185–196. 10.1534/genetics.105.046565 16079224 PMC1456146

[B21] Freeman M. R. (2015). *Drosophila* Central Nervous System Glia. Cold Spring Harb. Perspect. Biol. 7:a020552. 10.1101/cshperspect.a020552 25722465 PMC4632667

[B22] Freeman M. R. Delrow J. Kim J. Johnson E. Doe C. Q. (2003). Unwrapping glial biology: Gcm target genes regulating glial development, diversification, and function. Neuron 38, 567–580. 10.1016/S0896-6273(03)00289-7 12765609

[B23] Ganapathy V. Thangaraju M. Gopal E. Martin P. M. Itagaki S. Miyauchi S.. (2008). Sodium-coupled monocarboxylate transporters in normal tissues and in cancer. AAPS J. 10, 193–199. 10.1208/s12248-008-9022-y 18446519 PMC2751467

[B24] Gordon G. R. Choi H. B. Rungta R. L. Ellis-Davies G. C. MacVicar B. A. (2008). Brain metabolism dictates the polarity of astrocyte control over arterioles. Nature 456, 745–749. 10.1038/nature07525 18971930 PMC4097022

[B25] Gorska-Andrzejak J. Salvaterra P. M. Meinertzhagen I. A. Krzeptowski W. Gorlich A. Pyza E. (2009). Cyclical expression of Na+/K+-ATPase in the visual system of Drosophila melanogaster. J. Insect. Physiol. 55, 459–468. 10.1016/j.jinsphys.2009.02.003 19428365 PMC2721802

[B26] Hakim Y. Yaniv S. P. Schuldiner O. (2014). Astrocytes play a key role in Drosophila mushroom body axon pruning. PLoS ONE 9:e86178. 10.1371/journal.pone.0086178 24465945 PMC3897647

[B27] Haydon P. G. Nedergaard M. (2015). How do astrocytes participate in neural plasticity? Cold Spring Harb. Perspect. Biol. 7:a020438. 10.1101/cshperspect.a020438 25502516 PMC4355266

[B28] Heiman M. Schaefer A. Gong S. Peterson J. D. Day M. Ramsey K. E.. (2008). A translational profiling approach for the molecular characterization of CNS cell types. Cell 135, 738–748. 10.1016/j.cell.2008.10.028 19013281 PMC2696821

[B29] Huang D. W. Lempicki R. A. (2009). Systematic and integrative analysis of large gene lists using DAVID bioinformatics resources. Nat. Protoc. 4, 44–57. 10.1038/nprot.2008.211 19131956

[B30] Huang X. Warren J. T. Buchanan J. Gilbert L. I. Scott M. P. (2007). Drosophila Niemann-Pick type C-2 genes control sterol homeostasis and steroid biosynthesis: a model of human neurodegenerative disease. Development 134, 3733–3742. 10.1242/dev.004572 17804599

[B31] Huang Y. Ainsley J. A. Reijmers L. G. Jackson F. R. (2013). Translational profiling of clock cells reveals circadianly synchronized protein synthesis. PLoS Biol. 11:e1001703. 10.1371/journal.pbio.1001703 24348200 PMC3864454

[B32] Huang Y. McNeil G. P. Jackson F. R. (2014). Translational regulation of the DOUBLETIME/CKI delta/epsilon kinase by LARK contributes to circadian period modulation. PLoS Genet. 10:e1004536. 10.1371/journal.pgen.1004536 25211129 PMC4161311

[B33] Huang Y. Ng F. S. Jackson F. R. (2015). Comparison of larval and adult Drosophila astrocytes reveals stage-specific gene expression profiles. G3 (Bethesda.) 5, 551–558. 10.1534/g3.114.016162 25653313 PMC4390571

[B34] Ishimoto H. Kitamoto T. (2010). The steroid molting hormone Ecdysone regulates sleep in adult Drosophila melanogaster. Genetics 185, 269–281. 10.1534/genetics.110.114587 20215472 PMC2870962

[B35] Ishimoto H. Kitamoto T. (2011). Beyond molting–roles of the steroid molting hormone ecdysone in regulation of memory and sleep in adult Drosophila. Fly (Austin) 5, 215–220. 10.4161/fly.5.3.15477 21444997 PMC3225765

[B36] Itoh T. Q. Tanimura T. Matsumoto A. (2011). Membrane-bound transporter controls the circadian transcription of clock genes in Drosophila. Genes Cells 16, 1159–1167. 10.1111/j.1365-2443.2011.01559.x 22077638

[B37] Jackson F. R. Ng F. S. Sengupta S. You S. Huang Y. (2015). Glial cell regulation of rhythmic behavior. Meth. Enzymol. 552, 45–73. 10.1016/bs.mie.2014.10.016 25707272 PMC4662800

[B38] Jaumouillè E. Machado A. P. Stähli P. Koch R. Nagoshi E. (2015). Transcriptional regulation via nuclear receptor crosstalk required for the Drosophila circadian clock. Curr. Biol. 25, 1502–1508. 10.1016/j.cub.2015.04.017 26004759 PMC4454776

[B39] Kamae Y. Uryu O. Miki T. Tomioka K. (2014). The nuclear receptor genes HR3 and E75 are required for the circadian rhythm in a primitive insect. PLoS ONE 9:e114899. 10.1371/journal.pone.0114899 25502221 PMC4263706

[B40] Kucukdereli H. Allen N. J. Lee A. T. Feng A. Ozlu M. I. Conatser L. M.. (2011). Control of excitatory CNS synaptogenesis by astrocyte-secreted proteins Hevin and SPARC. Proc. Natl. Acad. Sci. U.S.A. 108, E440–E449. 10.1073/pnas.1104977108 21788491 PMC3156217

[B41] Kumar S. Chen D. Jang C. Nall A. Zheng X. Sehgal A. (2014). An ecdysone-responsive nuclear receptor regulates circadian rhythms in Drosophila. Nat. Commun. 5, 5697. 10.1038/ncomms6697 25511299 PMC4269253

[B42] Langmead B. Salzberg S. L. (2012). Fast gapped-read alignment with Bowtie 2. Nat. Methods 9, 357–359. 10.1038/nmeth.1923 22388286 PMC3322381

[B43] Lazareva A. A. Roman G. Mattox W. Hardin P. E. Dauwalder B. (2007). A role for the adult fat body in Drosophila male courtship behavior. PLoS Genet. 3:e16. 10.1371/journal.pgen.0030016 17257054 PMC1781494

[B44] Levine J. D. Funes P. Dowse H. B. Hall J. C. (2002). Signal analysis of behavioral and molecular cycles. BMC Neurosci. 3:1. 10.1186/1471-2202-3-1 11825337 PMC65508

[B45] Liu H. Zhou B. Yan W. Lei Z. Zhao X. Zhang K.. (2014). Astrocyte-like glial cells physiologically regulate olfactory processing through the modification of ORN-PN synaptic strength in *Drosophila*. Eur. J. Neurosci. 40, 2744–2754. 10.1111/ejn.12646 24964821

[B46] Matsuno M. Horiuchi J. Yuasa Y. Ofusa K. Miyashita T. Masuda T.. (2015). Long-term memory formation in Drosophila requires training-dependent glial transcription. J. Neurosci. 35, 5557–5565. 10.1523/JNEUROSCI.3865-14.2015 25855172 PMC6605317

[B47] McMahon H. T. Boucrot E. (2011). Molecular mechanism and physiological functions of clathrin-mediated endocytosis. Nat. Rev. Mol. Cell Biol. 12, 517–533. 10.1038/nrm3151 21779028

[B48] Melom J. E. Littleton J. T. (2013). Mutation of a NCKX eliminates glial microdomain calcium oscillations and enhances seizure susceptibility. J. Neurosci. 33, 1169–1178. 10.1523/JNEUROSCI.3920-12.2013 23325253 PMC3600868

[B49] Miller D. Hannon C. Ganetzky B. (2012). A mutation in Drosophila Aldolase causes temperature-sensitive paralysis, shortened lifespan, and neurodegeneration. J. Neurogenet. 26, 317–327. 10.3109/01677063.2012.706346 22882183 PMC3597222

[B50] Ng F. S. Jackson F. R. (2015). The ROP vesicle release factor is required in adult Drosophila glia for normal circadian behavior. Front. Cell Neurosci. 9:256. 10.3389/fncel.2015.00256 26190976 PMC4490253

[B51] Ng F. S. Tangredi M. M. Jackson F. R. (2011). Glial cells physiologically modulate clock neurons and circadian behavior in a calcium-dependent manner. Curr. Biol. 21, 625–634. 10.1016/j.cub.2011.03.027 21497088 PMC3081987

[B52] Okamoto N. Nishimura T. (2015). Signaling from Glia and Cholinergic neurons controls nutrient-dependent production of an Insulin-like Peptide for Drosophila body growth. Dev. Cell 35, 295–310. 10.1016/j.devcel.2015.10.003 26555050

[B53] Omoto J. J. Yogi P. Hartenstein V. (2015). Origin and development of neuropil glia of the Drosophila larval and adult brain: two distinct glial populations derived from separate progenitors. Dev. Biol. 404, 2–20. 10.1016/j.ydbio.2015.03.004 25779704 PMC4515183

[B54] Parpura V. Zorec R. (2010). Gliotransmission: exocytotic release from astrocytes. Brain Res. Rev. 63, 83–92. 10.1016/j.brainresrev.2009.11.008 19948188 PMC2862866

[B55] Petersen A. J. Rimkus S. A. Wassarman D. A. (2012). ATM kinase inhibition in glial cells activates the innate immune response and causes neurodegeneration in Drosophila. Proc. Natl. Acad. Sci. U.S.A. 109, E656–E664. 10.1073/pnas.1110470109 22355133 PMC3306708

[B56] Petersen T. N. Brunak S. von H. G. Nielsen H. (2011). SignalP 4.0: discriminating signal peptides from transmembrane regions. Nat. Methods 8, 785–786. 10.1038/nmeth.1701 21959131

[B57] Poitry-Yamate C. L. Poitry S. Tsacopoulos M. (1995). Lactate released by Muller glial cells is metabolized by photoreceptors from mammalian retina. J. Neurosci. 15, 5179–5191. 7623144 10.1523/JNEUROSCI.15-07-05179.1995 PMC6577914

[B58] Rahman M. Ham H. Liu X. Sugiura Y. Orth K. Kramer H. (2012). Visual neurotransmission in Drosophila requires expression of Fic in glial capitate projections. Nat. Neurosci. 15, 871–875. 10.1038/nn.3102 22544313 PMC3578554

[B59] Rapti G. Richmond J. Bessereau J. L. (2011). A single immunoglobulin-domain protein required for clustering acetylcholine receptors in C. elegans. EMBO J. 30, 706–718. 10.1038/emboj.2010.355 21252855 PMC3041951

[B60] Rival T. Soustelle L. Strambi C. Besson M. T. Iche M. Birman S. (2004). Decreasing glutamate buffering capacity triggers oxidative stress and neuropil degeneration in the Drosophila brain. Curr. Biol. 14, 599–605. 10.1016/j.cub.2004.03.039 15062101

[B61] Rouach N. Koulakoff A. Abudara V. Willecke K. Giaume C. (2008). Astroglial metabolic networks sustain hippocampal synaptic transmission. Science 322, 1551–1555. 10.1126/science.1164022 19056987

[B62] Rusan Z. M. Kingsford O. A. Tanouye M. A. (2014). Modeling Glial Contributions to Seizures and Epileptogenesis: Cation-Chloride Cotransporters in Drosophila melanogaster. PLoS ONE 9:e101117. 10.1371/journal.pone.0101117 24971529 PMC4074161

[B63] Schirmeier S. Matzat T. Klämbt C. (2016). Axon ensheathment and metabolic supply by glial cells in Drosophila. Brain Res. 1641(Pt A), 122–129. 10.1016/j.brainres.2015.09.003 26367447

[B64] Seal R. L. Gordon S. M. Lush M. J. Wright M. W. Bruford E. A. (2011). genenames.org: the HGNC resources in 2011. Nucleic Acids Res. 39, D514–D519. 10.1093/nar/gkq892 20929869 PMC3013772

[B65] Seugnet L. Suzuki Y. Merlin G. Gottschalk L. Duntley S. P. Shaw P. J. (2011). Notch signaling modulates sleep homeostasis and learning after sleep deprivation in Drosophila. Curr. Biol. 21, 835–840. 10.1016/j.cub.2011.04.001 21549599 PMC3741064

[B66] Singh S. K. Stogsdill J. A. Pulimood N. S. Dingsdale H. Kim Y. H. Pilaz L. J.. (2016). Astrocytes assemble thalamocortical synapses by bridging NRX1alpha and NL1 via Hevin. Cell 164, 183–196. 10.1016/j.cell.2015.11.034 26771491 PMC4715262

[B67] Stork T. Bernardos R. Freeman M. R. (2012). Analysis of glial cell development and function in Drosophila. Cold Spring Harb. Protoc. 2012, 1–17. 10.1101/pdb.top067587 22194269 PMC5193132

[B68] Stork T. Sheehan A. Tasdemir-Yilmaz O. E. Freeman M. R. (2014). Neuron-Glia Interactions through the Heartless FGF receptor signaling pathway mediate Morphogenesis of Drosophila Astrocytes. Neuron 83, 388–403. 10.1016/j.neuron.2014.06.026 25033182 PMC4124900

[B69] Strauss A. L. Kawasaki F. Ordway R. W. (2015). A distinct perisynaptic glial cell type forms tripartite neuromuscular synapses in the Drosophila adult. PLoS ONE 10:e0129957. 10.1371/journal.pone.0129957 26053860 PMC4459971

[B70] Suh J. Jackson F. R. (2007). Drosophila ebony activity is required in glia for the circadian regulation of locomotor activity. Neuron 55, 435–447. 10.1016/j.neuron.2007.06.038 17678856 PMC2034310

[B71] Tasdemir-Yilmaz O. E. Freeman M. R. (2014). Astrocytes engage unique molecular programs to engulf pruned neuronal debris from distinct subsets of neurons. Genes Dev. 28, 20–33. 10.1101/gad.229518.113 24361692 PMC3894410

[B72] Thomas A. Lee P. J. Dalton J. E. Nomie K. J. Stoica L. Costa-Mattioli M.. (2012). A versatile method for cell-specific profiling of translated mRNAs in Drosophila. PLoS ONE 7:e40276. 10.1371/journal.pone.0040276 22792260 PMC3391276

[B73] Trapnell C. Hendrickson D. G. Sauvageau M. Goff L. Rinn J. L. Pachter L. (2013). Differential analysis of gene regulation at transcript resolution with RNA-seq. Nat. Biotechnol. 31, 46–53. 10.1038/nbt.2450 23222703 PMC3869392

[B74] Trapnell C. Pachter L. Salzberg S. L. (2009). TopHat: discovering splice junctions with RNA-Seq. Bioinformatics 25, 1105–1111. 10.1093/bioinformatics/btp120 19289445 PMC2672628

[B75] Truman J. W. Talbot W. S. Fahrbach S. E. Hogness D. S. (1994). Ecdysone receptor expression in the CNS correlates with stage-specific responses to ecdysteroids during Drosophila and Manduca development. Development 120, 219–234. 8119129 10.1242/dev.120.1.219

[B76] Uttenweiler-Joseph S. Moniatte M. Lagueux M. Van D. A. Hoffmann J. A. Bulet P. (1998). Differential display of peptides induced during the immune response of Drosophila: a matrix-assisted laser desorption ionization time-of-flight mass spectrometry study. Proc. Natl. Acad. Sci. U.S.A. 95, 11342–11347. 10.1073/pnas.95.19.11342 9736738 PMC21644

[B77] Vance J. E. (2012). Dysregulation of cholesterol balance in the brain: contribution to neurodegenerative diseases. Dis. Model. Mech. 5, 746–755. 10.1242/dmm.010124 23065638 PMC3484857

[B78] Verkhratsky A. Matteoli M. Parpura V. Mothet J. P. Zorec R. (2016). Astrocytes as secretory cells of the central nervous system: idiosyncrasies of vesicular secretion. EMBO J. 35, 239–257. 10.15252/embj.201592705 26758544 PMC4741299

[B79] Volkenhoff A. Weiler A. Letzel M. Stehling M. Klambt C. Schirmeier S. (2015). Glial Glycolysis Is Essential for Neuronal Survival in Drosophila. Cell Metab. 22, 437–447. 10.1016/j.cmet.2015.07.006 26235423

[B80] Xu Y. An F. Borycz J. A. Borycz J. Meinertzhagen I. A. Wang T. (2015). Histamine recycling is mediated by CarT, a carcinine transporter in Drosophila Photoreceptors. PLoS Genet. 11:e1005764. 10.1371/journal.pgen.1005764 26713872 PMC4694695

[B81] Yamazaki D. Horiuchi J. Ueno K. Ueno T. Saeki S. Matsuno M.. (2014). Glial dysfunction causes age-related memory impairment in Drosophila. Neuron 84, 753–763. 10.1016/j.neuron.2014.09.039 25447741

[B82] Zhang Y. Chen K. Sloan S. A. Bennett M. L. Scholze A. R. O'Keeffe S.. (2014). An RNA-sequencing transcriptome and splicing database of glia, neurons, and vascular cells of the cerebral cortex. J. Neurosci. 34, 11929–11947. 10.1523/JNEUROSCI.1860-14.2014 25186741 PMC4152602

[B83] Zorec R. Horvat A. Vardjan N. Verkhratsky A. (2015). Memory formation shaped by Astroglia. Front. Integr. Neurosci. 9:56. 10.3389/fnint.2015.00056 26635551 PMC4648070

[B84] Zorec R. Verkhratsky A. Rodríguez J. J. Parpura V. (2016). Astrocytic vesicles and gliotransmitters: slowness of vesicular release and synaptobrevin2-laden vesicle nanoarchitecture. Neuroscience 323, 67–75. 10.1016/j.neuroscience.2015.02.033 25727638

[B85] Zwarts L. Van Eijs F. Callaerts P. (2015). Glia in *Drosophila* behavior. J. Comp. Physiol. A Neuroethol. Sens. Neural Behav. Physiol. 201, 879–893. 10.1007/s00359-014-0952-9 25336160

