# Supplementary figures and images for: TRAP-seq Profiling and RNAi-Based Genetic Screens Identify Conserved Glial Genes Required for Adult Drosophila Behavior
Source: Front Mol Neurosci. 2016 Dec 22;9:146. doi: 10.3389/fnmol.2016.00146 (PMC5177635; doi:10.3389/fnmol.2016.00146)

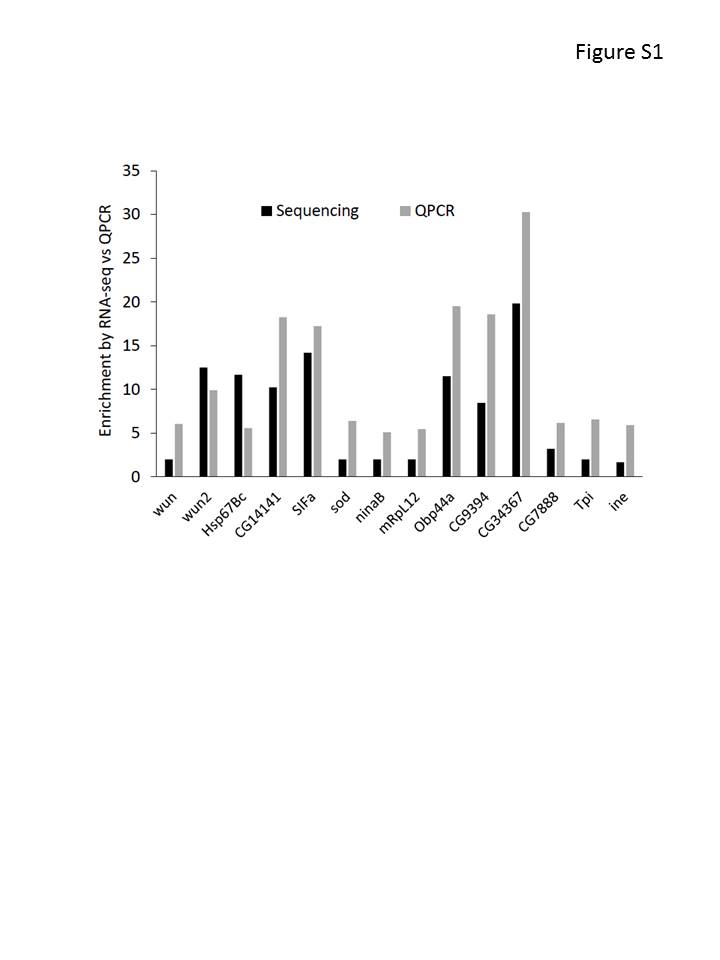

Supplement: Figure S1 — Fourteen genes with various FPKM values show astrocyte enrichment (TRAP compared to total RNA) by either RNA-seq or QPCR analysis. QPCR methods are described in the Section Materials and Methods. In the case of most genes showing greater than ~8-fold enrichment by RNA-seq (6 out of 7), a similar or higher enrichment value was observed with QPCR. In contrast, those genes showing lower enrichment by RNA-seq showed reduced enrichment when assayed by QPCR. [file Image1.jpeg]

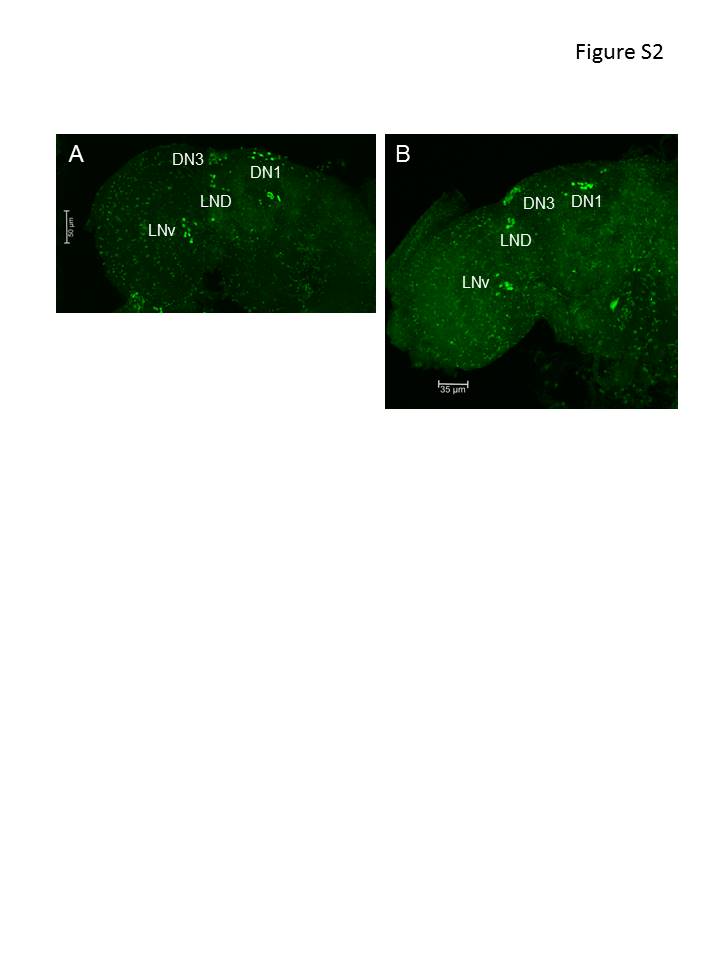

Supplement: Figure S2 — Knockdown of CG9657 did not affect the number of PER-containing cells. (A) control brain hemisphere, (B) repo-Gal4>CG9657.IR hemisphere. DN1, Dorsal neurons 1; DN3, Dorsal neurons 3; LNv, ventral lateral neurons; LND, Dorsal lateral neurons. Note the different scales for the hemispheres shown in (A,B). More than 30 brain hemispheres were examined for each genotype. [file Image2.jpeg]

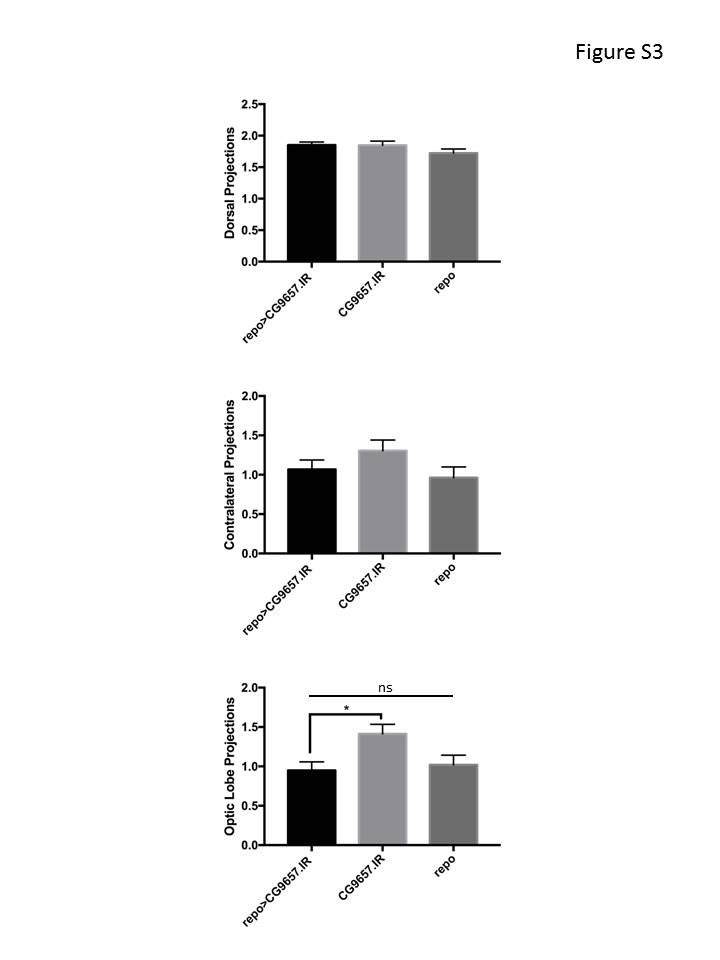

Supplement: Figure S3 — Quantification of PDF projection morphology in control brains and those with pan-glial expression of CG9657.IR (see Section Materials and Methods for details). In three biological replicates (two pooled in this figure), glial expression of CG9657.IR did not result in abnormal morphology for s-LNv dorsal projections (top), l-LNv contralateral (POT) projections (middle) or l-LNv optic lobe projections (bottom) in comparison to both UAS and GAL4 genetic controls. While there was altered morphology for optic lobe projections in CG9657.IR-expressing flies compared to the UAS control, there was no significant difference between experimental and Gal4 control brains. Results are mean ± SEM; n = 23–30 brain hemispheres; *p < 0.05 for a one-way ANOVA with Tukey's multiple comparison test; ns, not significant. [file Image3.jpeg]
