# Supplementary material for: TRAP-seq Profiling and RNAi-Based Genetic Screens Identify Conserved Glial Genes Required for Adult Drosophila Behavior
Source: Front Mol Neurosci. 2016 Dec 22;9:146. doi: 10.3389/fnmol.2016.00146 (PMC5177635; doi:10.3389/fnmol.2016.00146)
Supplement: Table S5 — Genes detected by TRAP profiling that are known to be expressed in fly glia. [file Table5.PDF]

Table S5. Genes detected by TRAP profiling that are known to be expressed in fly glia.

Fly Gene	Fold enriched	Protein	Function
CG1732 (GAT)	3.8	GABA transporter	Membrane GABA transport
CG7433	4.3	GABA transaminase	GABA inactivation
Gs2	3.9	Glutamine synthase 2	Glutamate neurotransmitter recycling
Got 1 and 2	2.6 (1)<br>1.4 (2)	Glutamate oxaloacetate transaminase	Glutamate metabolism
Dat	2.9	Dopamine acetyltransferase	Dopamine metabolism
aay	2.2	Astray	Serine metabolism
ine	1.5	Inebriated	Neurotransmitter transporter
Irk 2 and 3	2.6 (2)<br>12.3 (3)	Inwardly rectifying potassium channel	K^+ ion channel
CG9657	2.1	SLC5A transporter	Sodium/solute symporter
PC	NA	Pyruvate decarboxylase	Pyruvate/D-serine metabolism
